# TRIM29 negatively controls antiviral immune response through targeting STING for degradation

**DOI:** 10.1038/s41421-018-0010-9

**Published:** 2018-03-20

**Authors:** Qijie Li, Liangbin Lin, Yanli Tong, Yantong Liu, Jun Mou, Xiaodong Wang, Xiuxuan Wang, Yanqiu Gong, Yi Zhao, Yi Liu, Bo Zhong, Lunzhi Dai, Yu-Quan Wei, Huiyuan Zhang, Hongbo Hu

**Affiliations:** 1 Department of Rheumatology and Immunology, State Key Laboratory of Biotherapy and Collaborative Innovation Center for Biotherapy, West China Hospital, Sichuan University, Chengdu, 610041 China; 2 Department of General Practice and Lab of PTM, State Key Laboratory of Biotherapy and Collaborative Innovation Center for Biotherapy, West China Hospital, Sichuan University, Chengdu, 610041 China; 3 Cancer Center, State Key Laboratory of Biotherapy and Collaborative Innovation Center for Biotherapy, West China Hospital, Sichuan University, Chengdu, 610041 China; 4 School of Life Science, Wuhan University, Wuhan, China

## Abstract

Innate immune system is armed by several lines of pattern recognition receptors to sense various viral infection and to initiate antiviral immune response. This process is under a tight control and the negative feedback induced by infection and/or inflammation is critical to maintain immune homoeostasis and to prevent autoimmune disorders, however, the molecular mechanism is not fully understood. Here we report TRIM29, a ubiquitin E3 ligase, functions as an inducible negative regulator of innate immune response triggered by DNA virus and cytosolic DNA. DNA virus and cytosolic DNA stimulation induce TRIM29 expression robustly in macrophages and dendritic cells, although the basal level of TRIM29 is undetectable in those cells. TRIM29 deficiency elevates IFN-I and proinflammatory cytokine production upon viral DNA and cytosolic dsDNA stimulation. Consistently, in vivo experiments show that TRIM29-deficient mice are more resistant to HSV-1 infection than WT controls, indicated by better survival rate and reduced viral load in organs. Mechanism studies suggest that STING–TBK1–IRF3 signaling pathway in TRIM29 KO cells is significantly enhanced and the degradation of STING is impaired. Furthermore, we identify that TRIM29 targets STING for K48 ubiquitination and degradation. This study reveals TRIM29 as a crucial negative regulator in immune response to DNA virus and cytosolic DNA, preventing potential damage caused by overcommitted immune responses.

## Introduction

Antiviral immunity is initiated by the recognition of virus-derived nuclear acids via germline-encoded pattern recognition receptors (PRRs) of innate immune cells, which activates downstream signal pathways leading to production of type I interferon-I (IFN-I) and proinflammatory cytokine^1^. Several categories of PRRs have been reported to sense the extracellular, endosomal, and cytosolic viral infection. Viral RNA is recognized by Toll-like receptor 3 (TLR3)^2^, TLR7/8^3^, retinoic acid-inducible gene I (RIG-1), laboratory of genetics and physiology 2 (LGP2)^4,5^, and melanoma differentiation associated gene 5 (MDA5)^6^. Viral DNA, as well as the pathogenic self-DNA leaked from nucleus and mitochondria, is strong inducer of IFN-I production^7^. Many sensors of DNA have been discovered, among which TLR9 is the first identified DNA sensor for bacterial unmethylated CpG DNA in endosome^8^; cytosolic DNA is also recognized by absent in melanoma 2 (AIM2)^9^, DNA-dependent activator of IFN regulator factors (DAI)^10^, RNA polymerase III^11^, IFN-gamma inducible factor 16 (IFI16)^12^, DDX41^13^, DNA-dependent protein kinase (DNA-PK)^14^ and cyclic-GMP-AMP (cGAMP) synthase (cGAS)^15,16^. DAI, IFI16, DNA-PK, and cGAS interact with signal adaptor stimulator of IFN genes (STING, also called MITA), a transmembrane protein located in mitochondria and endoplasmic reticulum (ER)^17,18^. Most of identified DNA sensors bind DNA and trigger IFN-I production, except AIM2 that induces inflammasome activation and IL-1β and IL-18 maturation^9^. IFI16 is the only DNA sensor that has the capacity to induce both inflammasome activation and IFN-I production^19,20^.

Although many DNA sensors have been identified, STING is thought to be the central molecule controlling DNA sensing and signaling cascades^21^. Upon recognition of pathogenic DNA, cGAS catalyzes the synthesis of cGAMP, which functions as a second messenger to interact with STING. Activated STING mediates the recruitment and activation of TANK-binding kinase 1 (TBK1). TBK1 also recruits and phosphorylates IFN regulator factor 3 (IRF3), promoting IRF3 dimerization and translocation into nucleus, to initiate the transcription of IFN-α, and IFN-β. Nuclear factor-κB (NF-κB) pathway is also activated by TBK1 to trigger proinflammatory cytokine production. Given the pivotal roles of IFN-I and proinflammatory cytokine in host defense against viral infection, production of these cytokines have to be tightly regulated to prevent overcommitted immune response. Therefore, STING is the key point to determine the intensity and duration of antiviral immune response.

There are multiple post-translational modification mechanisms reported to regulate the fate of STING. Cyclic dinucleotides activate ULK1 (ATG1) to phosphorylate STING leading to STING degradation^22^. Emerging evidence reveals the important role of TRIM family, an E3 ubiquitin ligase family with 75 numbers in human, in immune response against viral infection^23^. TRIM32 and TRIM56 cause K63 ubiquitination of STING is critical for its activation^24,25^, as well as recent study showed that K27 ubiquitination of STING mediated by INSIGI-AMFR had the similar effect^26^. Moreover, RNF5 (RMA1) targets STING for K48 ubiquitination-dependent degradation and therefore downregulates antiviral immune response^27^. Besides ubiquitination, sumoylation is critical for stability and activation of STING and cGAS. The balance of sumoylation and de-sumoylation is controlled by E3 ligase TRIM38 and sumoylation protease SUMO1/Sentrin/SMT3-specific peptidase (Senp2)^28^. Above all mentioned, ubiquitination provides fine tunes of STING stability and activation, and more intensive studies are needed to dissect the function of TRIM proteins in certain scenario. Recent report indicates TRIM29 is specifically expressed in alveolar macrophage (AM) to regulate immune response in respiratory tract^29^, however, the full-scale function of TRIM29 in innate immunity is largely unknown.

In this study, we obtained the biochemical and genetic evidence that TRIM29 is an inducible negative regulator of host defense against DNA virus infection, and its function is not limited in respiratory tract. TRIM29 mRNA was robustly induced by double-strand DNA (dsDNA) stimulation in dendritic cells and macrophage. Deficiency of TRIM29 led to enhanced IFN-I and proinflammatory cytokine production upon cytosolic DNA stimulation. The physiological function of TRIM29 was estimated using HSV-1 infection model. Compared with wild-type mice, TRIM29-deficient mice were more resistant to HSV-1 infection. Phosphorylation of IRF3, IκBα, and TBK1 was also significantly enhanced in TRIM29-deficient cells upon dsDNA stimulation. More importantly, the degradation of STING protein was suppressed in TRIM29-deficient cell after dsDNA stimulation. Further biochemical experiments revealed that TRIM29 served as the ubiquitin E3 ligase to catalyze K48 ubiquitination of STING, which induced then STING degradation upon its activation. TRIM29 bound to STING via its C-terminal, which was required for the STING ubiquitination. Our data collectively reveal a novel regulating mechanism of STING-dependent signaling pathway and establish TRIIM29 as a negative regulator of antiviral immune response to maintain immune homoeostasis, which might provide a novel intervention venue to control viral infection in future.

## Results

### TRIM29 is induced by cytosolic dsDNA stimulation

**Fig. 1:**
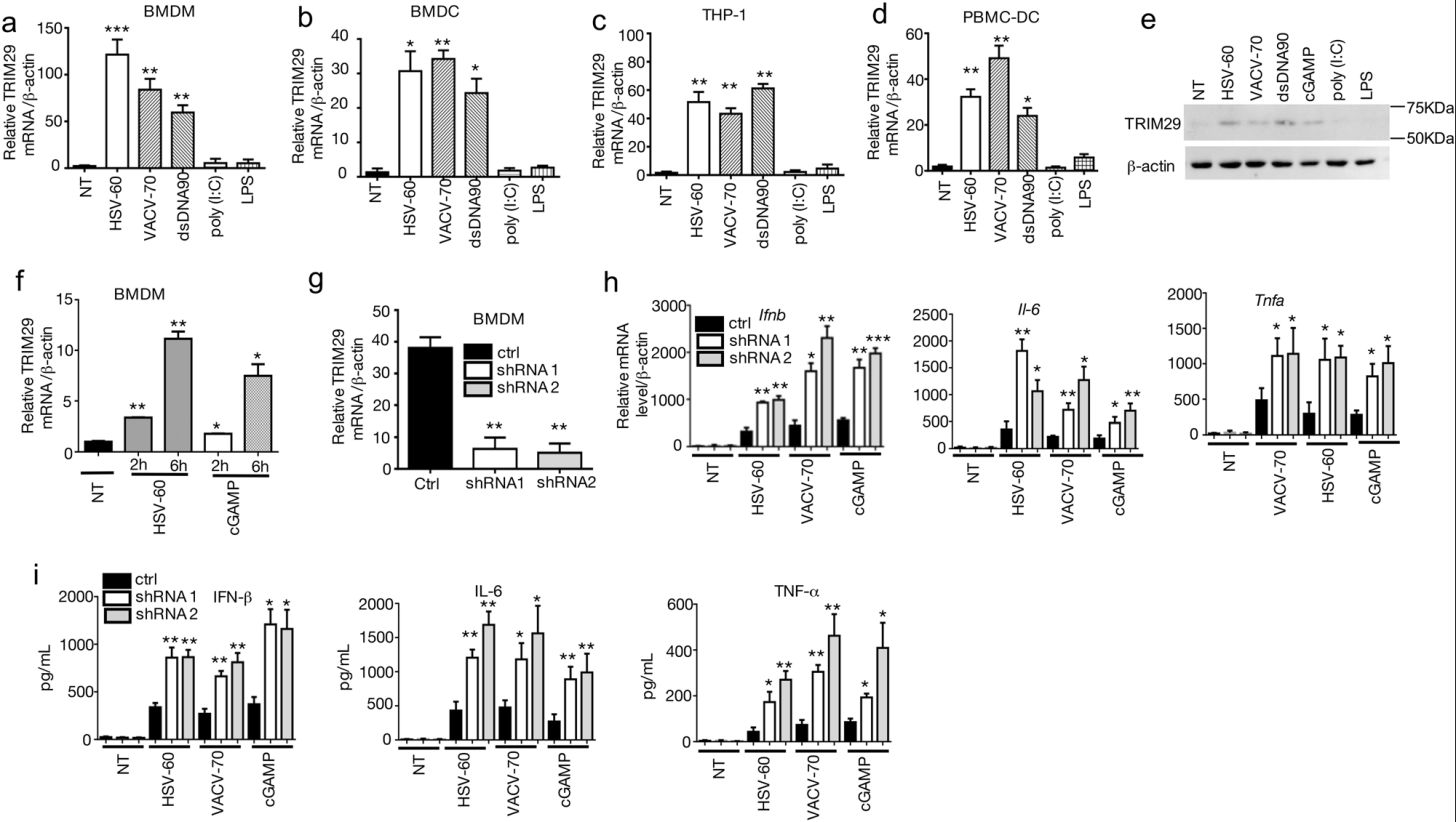
TRIM29 responses to cytosolic viral DNA stimulation and negatively regulates cytosolic DNA-induced cytokine production. Mouse BMDM **a** and BMDC **b**, human monocyte cell line THP-1 **c** and PBMC-derived DC **d** were stimulated with dsDNA90, VACV-70, HSV-60, LPS, and poly (I:C) for 12 h. The expression of TRIM29 mRNA was detected by real-time PCR and β-actin served as the reference gene. **e** BMDMs were treated with dsDNA90, VACV-70, HSV-60, cGAMP, LPS, and poly (I:C) for 24 h and protein level of TRIM29 was detected by IB using anti-TRIM29 antibody, β-actin served as the loading control. **f** BMDMs were treated with HSV-60 or cGAMP for 0, 2, or 6 h. The expression of TRIM29 mRNA was detected by real-time PCR and β-actin served as the reference gene. Two TRIM29-targeting shRNA (shRNA#1 and shRNA#2) and scramble shRNA (ctrl) were induced to mouse BMDM to knockdown TRIM29. The efficiency of knockdown was detected by real-time PCR **g**. BMDMs transfected with scramble shRNA or two TRIM29-targeting shRNA were stimulated with VACV-70, HSV-60, and cGAMP for 12 h, followed by real-time PCR assay **h** and ELISA **i** to detect the cytokine production. Data in all panels are representative of two to three independent experiments. *P < 0.05, **P < 0.01 and ***P < 0.001 (Student’s *t-*test). Error bars are s.d.

TRIM29 is recently reported to express highly in AM, which plays an nonredundant role in host defense against *Haemophilus influenza* infection and lipopolysaccharide (LPS) induced septic shock, as well as RNA virus infection^29^. However, the overall functions of TRIM29 in other types of innate immune cells and in other types of viral infection are largely unknown. As the TRIM29 expression level is very low in the unstimulated macrophage or dendritic cell (DC)^29^, we first examined whether TRIM29 expression level was induced by PAMP molecules, especially the pathogen-associated molecular pattern (PAMP) molecules derived from virus. We stimulated primary mouse bone marrow-derived macrophage (BMDM) and bone marrow-derived dendritic cell (BMDC) with LPS, poly (I:C) and several cytosolic DNA stimuli derived from herpes simplex virus type 1 (HSV-60), and vaccinia virus (VACV-70), and dsDNA (dsDNA90) delivered by lipofectamine 2000 for 12 h, followed by real-time PCR assay to detect TRIM29 mRNA level. Consistent with previous report^29^, TRIM29 mRNA level was very low in unstimulated BMDM and BMDC (Figs. 1a, b, non-treated samples). Interestingly, TRIM29 expression was strikingly upregulated by all cytosolic DNA stimulation (Figs. 1a, b). Neither TLR4 ligand LPS nor TLR3 ligand poly (I:C) induced TRIM29 expression. This phenotype was not limited in murine cells. Similarly, in human monocyte cell line THP-1 and peripheral blood mononuclear cell (PBMC)-derived dendritic cells, TRIM29 expression was also induced by HSV-60, VACV-70, dsDNA90, but not by LPS or poly (I:C) (Figs. 1c, d). Immunoblot (IB) results with anti-TRIM29 antibody confirmed that TRIM29 protein level was induced by cytosolic dsDNA stimuli, but not by LPS or poly (I:C) (Fig. 1e). Moreover, TRIM29 mRNA level was induced as early as 2 h after HSV-60 or cGAMP stimulation in BMDMs (Fig. 1f). These results indicate that cytosolic dsDNA stimulation induces TRIM29 expression in innate immune cells other than AM.

### TRIM29 mediates a negative feedback mechanism upon dsDNA stimulation

IFN-stimulated genes (ISGs) execute antiviral functions through multiple mechanisms including blocking virus replication and promoting antiviral cytokine production. Meanwhile, some ISGs also mediate negative feedbacks to dampen the overwhelming immune response^30,31^. The induction of TRIM29 expression by DNA virus suggested a potential role of TRIM29 in regulating antiviral immune response. To test our hypothesis, two TRIM29-targeting short hairpin RNAs (shRNAs) along with a control shRNA, were induced into mouse BMDMs. These cells were further stimulated with HSV-60, VACV-70, and cGAMP (a ligand of STING) delivered by Lipofectamine 2000 for 12 h. The knockdown efficiency was examined by quantitative PCR (qPCR) and IB. Compared with the control shRNA, TRIM29-specific shRNA significantly reduced TRIM29 expression in BMDMs (Fig. 1g). More importantly, knocking down TRIM29 led to a remarkably elevated IFN-β expression (Figs. 1h, i), as well as proinflammatory cytokine tumor necrosis factor (TNF)-α and interleukin-6 (IL-6). These data suggested that TRIM29 played a negative role in regulating dsDNA-induced IFN-I and proinflammatory cytokine production.

### TRIM29 restrains host defense against DNA viral infection in vivo

**Fig. 2:**
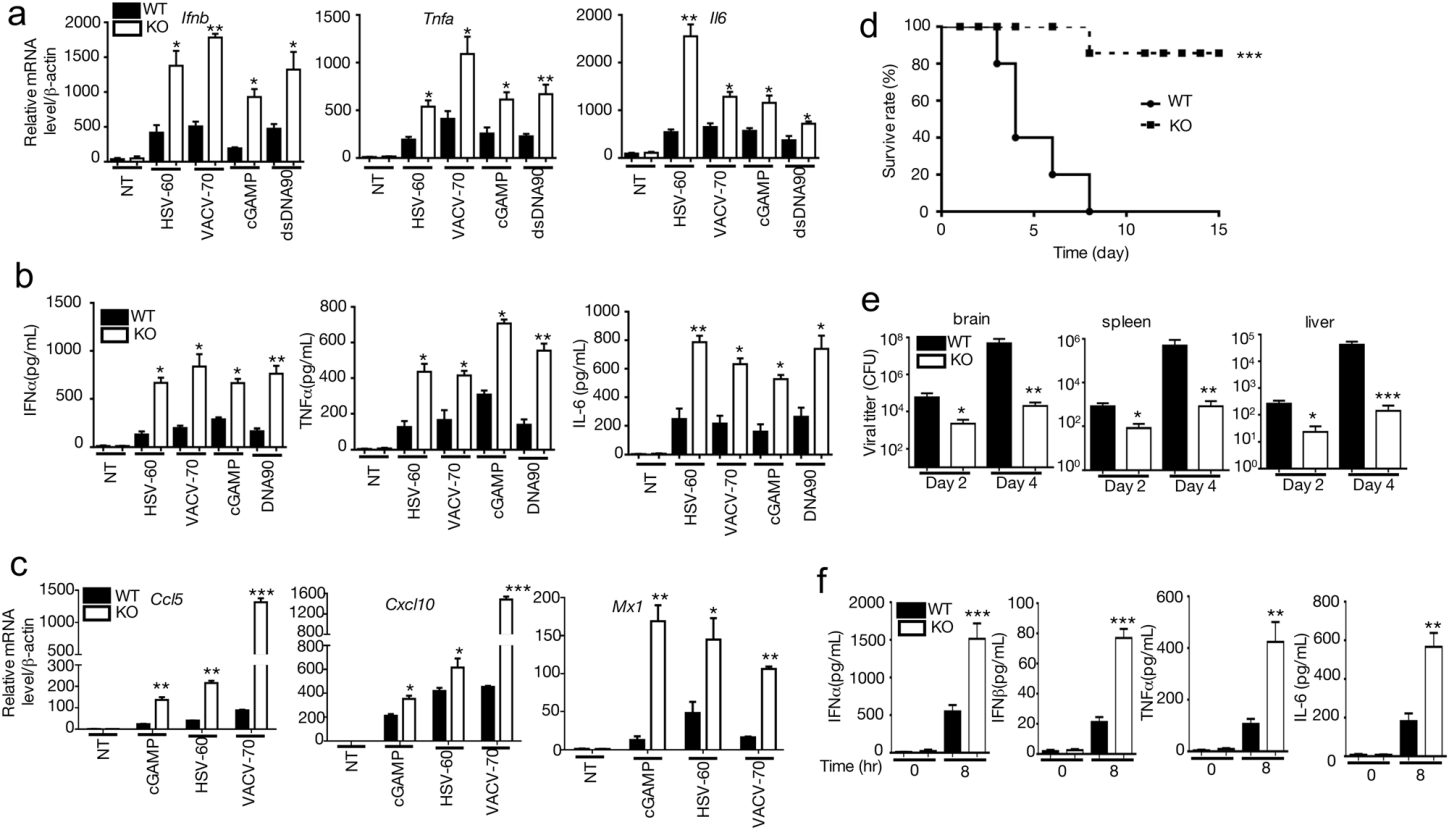
TRIM29 regulates host immune response against viral infection in vivo. Real-time PCR assay **a, c** and ELISA **b** were used to detect the expression of cytokine, chemokine and antiviral protein in WT and *Trim29*^−/−^ BMDMs stimulated with HSV-60, VACV-70, cGAMP, and DNA90 for 12 h. **d** WT and *Trim29*^*-/-*^ mice (*n* = 8 per strain) were infected by intravenous injected HSV-1 (2 × 10^7^ PFU per mouse) and monitored daily for 2 weeks for survival rate. **e** Viral titers of brains, livers and spleens from WT and *Trim29*^−/−^ mice (*n* = 3 per strain) 2 and 4 days after HSV-1 infection. **f** ELISA analysis of IFN-α, IFN-β, TNF-α and IL-6 in sera from WT and *Trim29*^−/−^ mice (*n* = 3 per strain) after HSV-1 infection. Data in all panels are representative of three independent experiments. *P < 0.05, **P < 0.01 and ***P < 0.001 (Student’s *t*-test). Error bars are s.d.

To study the physiological function of TRIM29, we generated TRIM29 knockout (*Trim29*^*-/-*^) mice using CRISPR-Cas9 technique. The schematic diagram of knockout strategy was shown in Supp. figure 1a. The knockout deficiency of TRIM29 was confirmed in the BMDMs (Supplementary Fig. 1b–1d). Similarly, as we observed above, HSV-60 dramatically induced TRIM29 expression in BMDMs from wild-type (WT) mice, but not in those from *Trim29*^*-/-*^ mice. Meanwhile, all the stimuli, including VACV-70, HSV-60, dsDNA, and cGAMP, significantly enhanced IFN-I, as well as proinflammatory cytokine production in TRIM29-deficient macrophage while compared with control (Figs. 2a, b), recapitulating what we observed when knocking down TRIM29. Furthermore, TRIM29 deficiency had little effect on poly (I:C)- or LPS-induced cytokine production (Supp. Fig. 3). In responses to VACV-70, HSV-60, dsDNA, and cGAMP*, Trim29*^*-/-*^ macrophages also produced significantly elevated CCL5 and CXCL10, the chemokines that are important to recruit immune cells to the site of infection and eliminate viral infection^32^. MX1 (also named IFI-78K, IFN-induced protein P78), a critical antiviral protein produced by host cells to antagonize the replication process of several different RNA and DNA viruses^33^, is highly induced in *Trim29*^-/-^ cells as well upon cGAMP, HSV-60, and VACV-70 stimulation (Fig. 2c). Taken together, these data indicated a negative role of TRIM29 in sensing viral dsDNA and cGAMP in BMDMs.

To further examine the function of TRIM29 in anti-DNA virus infection in vivo, we utilized an HSV-1 infection model. Age- and gender-matched WT and *Trim29*^-/-^ mice were infected with HSV-1 virus intravenously, and monitored daily for 2 weeks. All HSV-1-infected WT mice died in the first 10 days, however, most of infected *Trim29*^*-/-*^ mice survived during the 2-week monitor window, suggesting *Trim29*^*-/-*^mice were highly resistant to HSV-1 infection (Fig. 2d). Consistently, less HSV-1 virus was recovered in the brains, livers, and spleens isolated from *Trim29*^-/-^ mice 2 and 4 days after infection, respectively, compared with WT controls (Fig. 2e). IFN-α and IFN-β, together with proinflammatory cytokines, are critical for host antiviral response. *Trim29*^-/-^ mice had elevated levels of IFN-α and IFN-β in sera than WT controls after HSV-1 infection (Fig. 2f). We also observed higher IL-6 and TNF-α levels in sera isolated from *Trim29*^*-/-*^ mice than in those from WT mice (Fig. 2f). These data indicated that TRIM29 deficiency protected mice from DNA virus infection, therefore suggested that TRIM29 plays a critical role in dampening anti-DNA virus immune response.

### TRIM29 negatively regulates dsDNA-activated signaling pathway

**Fig. 3:**
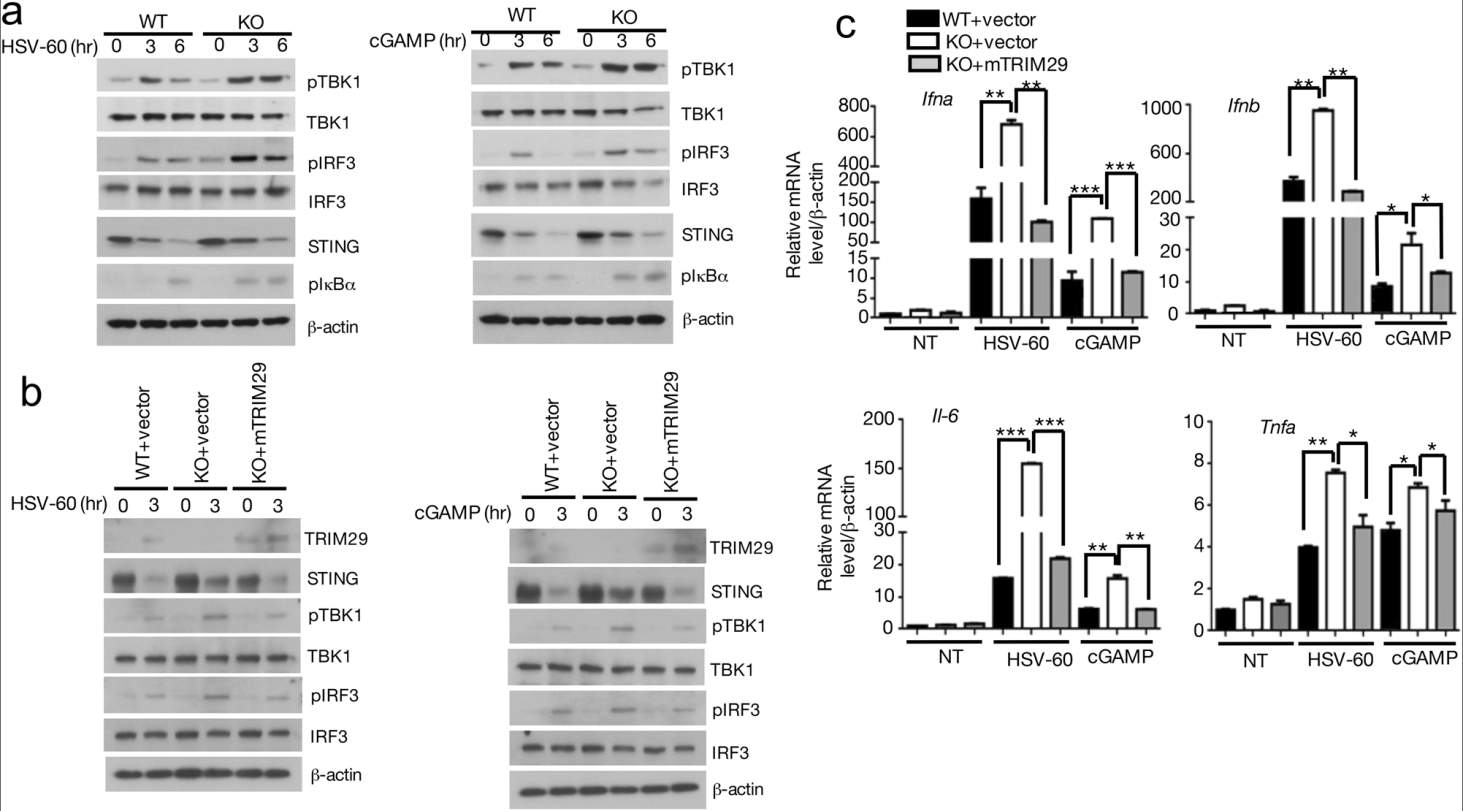
TRIM29 negatively regulates dsDNA-activated signaling pathway. **a** WT and *Trim29*^−/−^ BMDMs were stimulated with cGMAP and HSV-60 for indicated time, the cells were lysed and subjected to IB assay to detect phosphorylation of TBK1, IRF3, IκBα, and the degradation of STING. The total proteins or β-actin served as loading control. **b** WT, *Trim29*^-/-^, and TRIM29-reconstructed MEFs were stimulated as above for indicated time, and IB was performed to detect TRIM29, STING, phosphorylation of TBK1, IRF3. **c** These cells were stimulated as above for 12 h, and IFN-α, IFN-β, TNF-α, and IL-6 was detected by real-time PCR assay

Several DNA sensors, such as AIM2, IFI16, DDX41, and cGAS, have been discovered to initiate antiviral signal transduction. cGAS catalyzes the synthesis of cyclic GAMP from ATP and GTP in the cells exposed to dsDNA and DNA viruses. cGAMP binds to STING and activates STING–TBK1–IRF3 signaling pathway, to induce IFN-I and proinflammatory cytokine production. To detect whether TRIM29 was involved in this pathway, BMDMs from WT and *Trim29*^-/-^ mice were stimulated with STING ligand cGAMP and HSV-60, followed by IB to detect the activation of pathway by using antibodies specific for phosphorylated IRF3, IκBα, and TBK1. The results showed that phosphorylation of IRF3, IκBα, and its upstream kinase TBK1 was significantly enhanced in *Trim29*^-/-^ macrophage upon cGAMP and HSV-60 stimulation (Fig. 3a, Supp. Fig. 4), meanwhile the total TBK1 and IRF3 level remained the same in WT and *Trim29*^-/-^ cells. To further confirm the function of TRIM29 in dsDNA-induced immune response, TRIM29 was reconstituted into *Trim29*^-/-^ mouse embryonic fibroblasts (MEFs) (Supp. Fig. 5). TRIM29 reconstitution abolished the enhanced activation of STING–TBK1–IRF3 pathway in *Trim29*^-/-^MEF when stimulated by cGAMP and HSV-60 (Fig. 3b, Supp. Fig. 6), indicated by reduced phosphorylation of TBK1 and IRF3. Furthermore, reconstitution of TRIM29 into *Trim29*^*-/-*^ MEFs dramatically reduced IFN-I and proinflammatory cytokine production compared with *Trim29*^-/-^MEFs infected with control vector, restoring to the level observed in WT MEFs when challenged with HSV-60 and cGAMP (Fig. 3c). These data suggest that TRIM29 is a key negative regulator of STING–TBK1–IRF3 pathway.

### TRIM29 interacts with STING and regulates STING stability

**Fig. 4:**
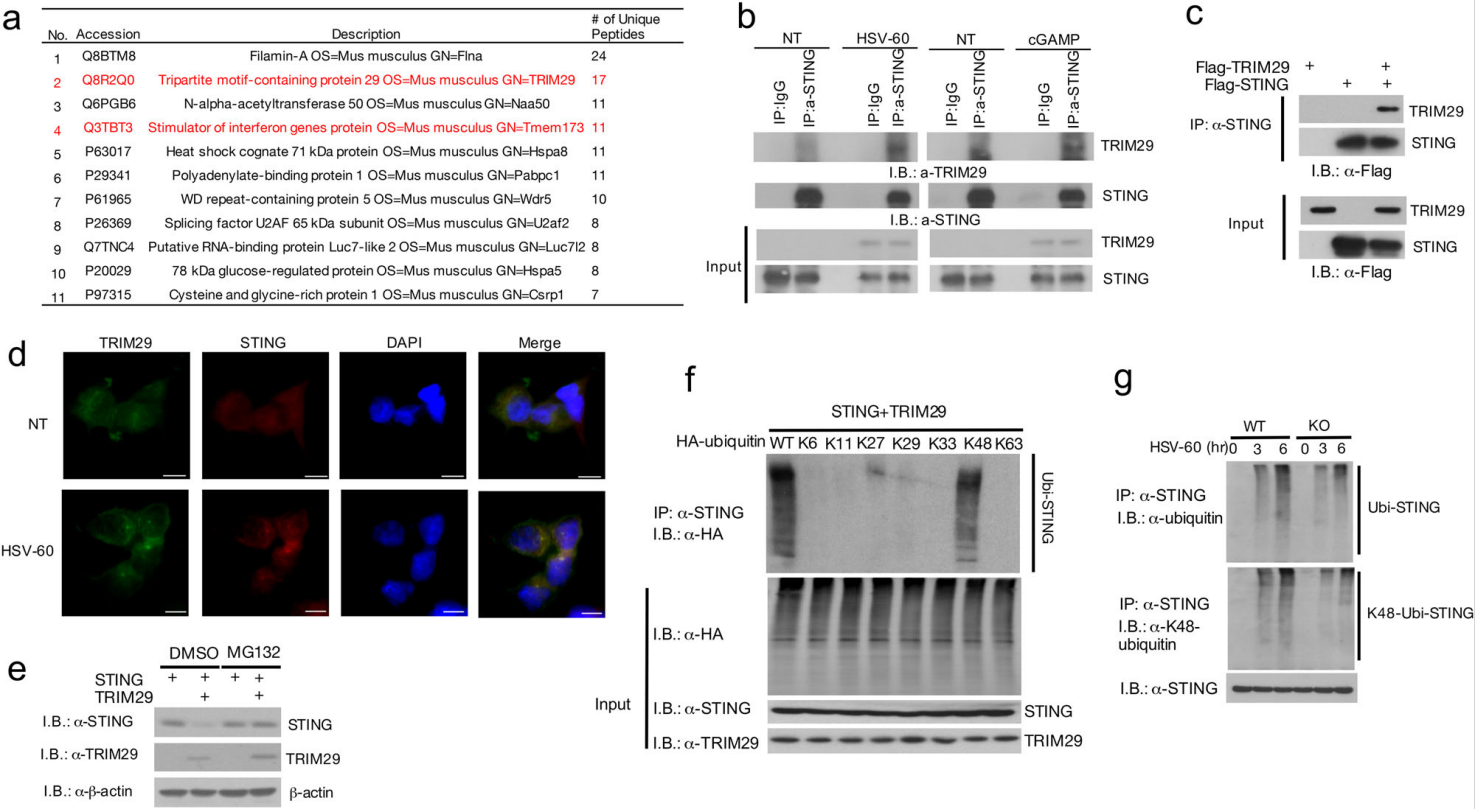
TRIM29 interacts with STING and mediates K48 linked ubiquitination and degradation of STING. **a** TRIM29-interacting proteins were identified by mass spectrum. Interaction between TRIM29 and STING was detected by co-immunoprecipitation (co-IP) in HSV-60 or cGMAP-stimulated BMDMs **b** and in the HEK 293T cell overexpressed TRIM29 and STING **c**. **d** Confocal microscopy of HEK 293T cells co-expressed Flag-TRIM29 and STING, non-treated and stimulated with HSV-60 for 6 h, followed by staining for Flag-TRIM29 (green) and STING (red). The bar in the picture stood for 1 μm. **e** HEK 293T cells were transfected with STING with or without TRIM29, treated with DMSO or MG132 for 12 h. STING and TRIM29 protein level was detected by IB. **f** HEK 293T cells were transfected with STING, TRIM29 and HA-tagged WT or mutant ubiquitin and subjected to ubiquitin assay. The ubiquitination of STING was detected by IB using anti-HA antibody. **g** WT and *Trim29*^*-/-*^ macrophages were stimulated with HSV-60 for indicated time. The cells were lysed and subjected to IP with anti-STING antibody, the ubiquitination of STING was detected by IB using anti-ubiquitin and anti-K48 ubiquitin antibody. Data in all panels (except panel **a**) are representative of three independent experiments

The protein level of STING is tightly controlled by a phosphorylation- and ubiquitination-dependent degradation upon viral infection^22,28^. Therefore, elevated TBK1, IRF3, and IκBα activation in TRIM29-deficient BMDM prompted us to further examined STING protein level. In line with previous observation, upon cGAMP and HSV-60 stimulation, STING protein level was significantly reduced in WT cells, however, the degradation of STING protein was impaired in KO cells (Fig. 3a). To probe the mechanism of how TRIM29 played a role in this signal pathway, we employed immunoprecipitation and mass spectrum (IP-MS) analysis to identify TRIM29-interacting proteins. Raw cells were stimulated by HSV-60 for 12 h with MG132 to block the proteasome-dependent protein degradation. The TRIM29-interacting proteins were isolated by IP using anti-TRIM29 antibody and identified by MS. STING (Tmem173) was among the identified TRIM29-interacting protein by IP-MS (Fig. 4a). Given that STING is a critical adaptor and sensor in dsDNA-mediated signaling pathways, and our previous data indicated that TRIM29 deficiency enhanced dsDNA-induced signaling cascades, we hypothesized that STING might be a direct target of TRIM29. To test our hypothesis, we examined the interaction of TRIM29 and STING in HSV-60 and cGMAP-stimulated BMDMs. Consistent with previous data, TRIM29 level was induced by dsDNA stimulation and formed a complex with STING after 24-h stimulation (Fig. 4b, Supp. Fig. 7a). This result was further confirmed by experiment in which overexpressed TRIM29 was strongly associated with STING (Fig. 4c, Supp. Fig. 7b). To examine the colocalization and subcellular localization of TRIM29 and STING, we overexpressed Flag-TRIM29 and HA-STING in HEK 293T cells followed by immunofluorescence staining. Under the unstimulated condition, both TRIM29 and STING were distributed in the cytosol (Fig. 4d, NT samples). However, in response to HSV-60 stimulation, STING aggregated perinuclearly and TRIM29 was found to colocalize with STING (Fig. 4d, HSV-60 stimulated samples). Taken together, our data indicate that TRIM29 interacts with STING and controls STING protein stability, leading to negative regulation of STING-dependent signaling pathway.

### TRIM29 mediates K48 ubiquitination of STING

As TRIM29 is an ubiquitin E3 ligase, which interacts with STING and regulates STING protein stability upon dsDNA stimulation, we hypothesized that TRIM29 might serve as the E3 ligase to induce K48 ubiquitination of STING and proteasome-dependent protein degradation. To examine our hypothesis, we overexpressed STING with or without TRIM29 in HEK 293T cells. Twelve hours before harvesting cells, MG132 was added to culture medium to block the proteasome-dependent protein degradation, DMSO was used as vehicle control. As we observed previously, co-expression of TRIM29 significantly reduced STING protein level (Fig. 4e); meanwhile MG132 treatment rescued the reduction of STING protein level mediated by TRIM29 (Fig. 4e, Supp. Fig. 8), suggesting TRIM29 regulates STING protein degradation in the proteasome-dependent manner.

The type of ubiquitination of STING decides the fates of STING, either activation or degradation. To elucidate the ubiquitination type of STING mediated by TRIM29, we overexpressed TRIM29, STING, and WT or mutant ubiquitin in single lysine (K6, K11, K11, K27, K29, K33, K48, and K63) into HEK 293T cells. After 12-h treatment with 10 μM MG132, the transfected cells were collected for ubiquitination assay. As shown in Fig. 4f, overexpression of WT and K48 ubiquitin, together with TRIM29 resulted in ubiquitination of STING. Other mutated ubiquitin failed to induce ubiquitination of STING, suggesting TRIM29 specifically mediated K48 ubiquitination of STING. This result was further confirmed at the endogenous level. WT and *Trim29*^-/-^ BMDMs were stimulated with HSV-60 for 3 and 6 h and the ubiquitination of STING was detected by IP-IB. Consistent with previous reports^26^, ubiquitination of STING was induced by HSV-60 stimulation (Fig. 4g), which was markedly reduced in *Trim29*^-/-^ macrophage, indicated by IB with anti-ubiquitin antibody. Furthermore, the IB with anti-K48 ubiquitin antibody indicated that the ubiquitination type of STING regulated by TRIM29 was K48 ubiquitination (Fig. 4g). Collectively, these data suggest TRIM29 serves as an E3 ligase for K48 ubiquitination of STING for degradation.

### TRIM29 interact with STING through C-terminal

**Fig. 5:**
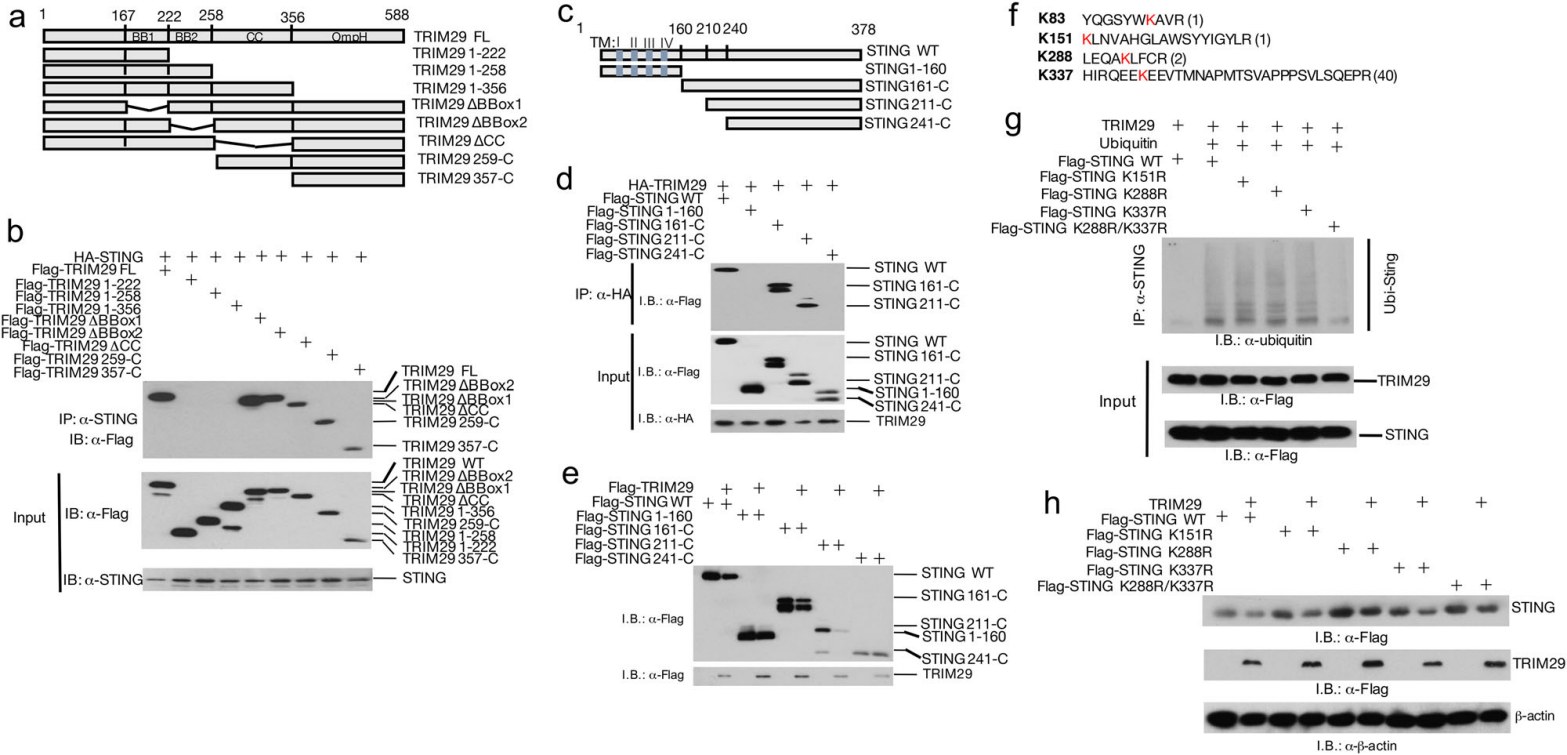
Identification of critical domains mediating TRIM29–STING interaction. **a** The schematic structure of TRIM29. **b** Full-length TRIM29 and different truncated TRIM29 mutants were co-expressed with STING in HEK 293T cells, and interaction between STING with WT or truncated TRIM29 was detected using IP and IB with indicated antibodies. **c** The schematic structure of STING. **d** The full-length STING and different truncated STING mutants were co-expressed with TRIM29, the interaction between TRIM29 with WT or truncated STING was detected by IP and IB with indicated antibodies. **e** The full-length STING and different truncated STING mutants were co-expressed with TRIM29 in HEK 293T cells and the protein level of STING (FL and truncated) and TRIM29 was detected with IB. **f** The Lys residues in STING were identified as potential targets of TRIM29 using IP-MS, highlighted in red. The number was frequency of specific peptides identified in IP product. **g** WT and mutated STING was co-expressed with TRIM29 and ubiquitin, the ubiquitination of STING was detected by IP and IB, and the protein level of STING was detected by IB, using indicated antibodies **h**

TRIM family members have relatively conserved N-terminal domains and diverse C-terminals. Therefore, it has been reported that C-terminals are responsible for interaction of TRIMs and their specific subtracts^23^. To map the crucial domain for STING and TRIM29 interaction, we generated different truncations of TRIM29 (1-222, 1-258, 1-356, ΔBBox1, ΔBBox2, ΔCC, 259-C, and 357-C; Fig. 5a), and overexpressed STING with full-length (FL) or truncated TRIM29 in HEK 293T cells followed by IP analysis. The mapping result suggested that FL TRIM29, together with ΔBBox1, ΔBBox2, ΔCC, and C-terminal of TRIM29 bound to STING, in contrast, N terminal of TRIM29, (1-222, 1-258, and 1-356) failed to interact with STING (Fig. 5b). These data suggest C-terminal of TRIM29 is crucial for STING–TRIM29 interaction.

As the key regulatory molecule in immune response to dsDNA virus infection, STING has been intensively studied and the crystal structure of STING protein has been illuminated^21^. In the N-terminal of STING, there are four transmembrane domains (TFs), which anchor STING in ER. To examine the domain of STING mediating STING–TRIM29 interaction, we also generated truncated STING (STING 1-160, STING 161-C, STING 211-C, and STING 241-C; Fig. 5c), and co-expressed with TRIM29 in HEK 293T cells, followed by IP to study the interaction of WT or truncated STING with TRIM29. Neither STING-160 nor STING (241-C) bound to TRIM29, whereas WT STING, STING 161-C, and STING 211-C formed a complex with TRIM29 (Fig. 5d). These data suggest that transmembrane domain or C-terminal of STING is not involved in STING–TRIM29 interaction. We further investigated which fragment(s) of STING respond to TRIM29-mediated protein degradation. The result indicated that STING FL, STING 161-C, and STING 211-C were degraded by co-expressed TRIM29; however, STING 1-160 and STING 241-C were resistant to TRIM29-mediated degradation (Fig. 5e), suggesting the TRIM29–STING interaction was required for TRIM29-mediated STING degradation.

Our data suggested that TRIM29 regulated degradative ubiquitination of STING; we employed IP-MS analysis to identify the ubiquitination acceptor site(s) of STING. The results revealed that four Lys residues, K83, K151, K288, and K337 might be regulated by TRIM29 (Supp. Fig. 9), although the K377 might be the major ubiquitination site as its unique peptide has been detected 40 times during MS assay (Fig. 5f). Single mutations of either those Lys residues to Args had little effect on the ubiquitination of STING mediated by TRIM29; however, mutation of two sites (K288R/K377R) generated a STING mutant that was defective in both ubiquitination and degradation caused by TRIM29 (Figs. 5g, h, Supp. Fig. 10). Our findings implicate that TRIM29 targets K288 and K337 for ubiquitination and degradation.

## Discussion

The data presented in this study identified TRIM29 as an inducible negative regulator of innate immune response against DNA virus and cytosolic dsDNA, preventing excessive immune response. In BMDC and BMDM, TRIM29 was significantly induced by dsDNA stimulation although the basal mRNA level of TRIM29 was undetectable. shRNA-mediated TRIM29 knockdown led to the enhanced IFN-I and proinflammatory cytokine production in response to cytosolic DNA. Consistently, the primary TRIM29-deficient BMDM produced more IFN-I, TNF-α, and IL-6, as well as chemokines and anti-virus protein, upon cytosolic dsDNA and cGAMP stimulation. In HSV-1 infection model, *Trim29*^*-/-*^ mice were resistant to virus infection, indicated by better survival rate and less viral load in organs, with higher IFN-I level in sera. Mechanism studies indicated that TRIM29 regulated STING–TBK1–IRF3 signaling pathway. IP-MS assay identified STING as a TRIM29-interacting protein, which was confirmed in the overexpression system and endogenous level. Ubiquitination assay suggested that TRIM29 functioned as the E3 ligase mediating K48 ubiquitination and degradation of STING. Further experiments showed that the C-terminal of TRIM29 was required for TRIM29–STING interaction; the transmembrane domain or C-terminal of STING was dispensable for TRIM29–STING interaction. K288 and K337 in STING were targeted by TRIM29 for ubiquitination and degradation. Our data elucidate that TRIM29 mediates a feedback mechanism that targets STING for its degradation and downregulation of antiviral immune response to prevent overwhelming immune pathogenesis.

As the key cytokine in antiviral immune response, IFN-I is quickly and robustly induced in many cell types after virus infection, however, host has developed many strategies to prevent overproduction of IFN-I and related harmful effects during acute infection. One mechanism involves inhibitory factors, such as ISG56, induced by viral infection to disrupt the key complex of IFN-I signaling pathway^30^. Another mechanism relies on the ubiquitin-dependent protein degradation. A line of E3 ubiquitin ligase family has been identified to target virus-induced IFN-I signaling pathways for degradation^26–29,33–35^. STING has been established as the key player in the anti-virus infection response, as its function is far beyond being a sensor, but also as important convergence to orchestrate signals from different DNA sensors^10,12–15^. The post-translational modifications, especially ubiquitination and sumoylation are essential for either activation or degradation of STING^28,34^, and the spectrum of STING-modifying enzymes is expending. Phosphorylation of STING by ATG1 reduces STING protein stability, which is critical to prevent sustained antiviral immune response^22^. Other modification, such as sumoylation of STING and c-GAS (controlled by TRIM38 and Senp2) is the mechanism to fine tune cGAS–STING pathway^28^. For ubiquitination, TRIM56 and TRIM32 promote K63-linked ubiquitination of STING that is required for STING activation. Besides K63 ubiquitination, K27 ubiquitination mediated by AMFR and INSIG1 complex and K11 ubiquitination mediated by RNF26, are also important to recruit TBK1 to STING for activating downstream signal^34^. On the other hand, it has been reported that RNF5 and TRIM30α induce K48-linked polyubiquitination that is responsible for STING degradation to negative regulate antiviral immune response^27,35^. Adding to these important findings, our data suggest that TRIM29 is inducible by DNA virus infection, also targeting STING for K48 ubiquitination and degradation. Zhong’s work^27^ reveals that RNF5 is an ER protein, suppressing immune response against RNA virus infection, meanwhile the expression of RNF5 is constitutive, but not induced by RNA virus. On the contrary, TRIM29 basal level is undetectable in unstimulated BMDCs and BMDMs, and is robustly induced by dsDNA stimulation to mediate STING degradation. Moreover, TRIM29 targets STING at K288 and K337, whereas RNF5 targets K150 for ubiquitination. Taken together, TRIM29 and RNF5 might regulate STING-dependent signaling pathway by different mechanisms. Recent study indicates that TRIM29 specifically expresses in AM and regulates anti-*H. influenza* infection and LPS-induced septic shock by targeting NEMO for ubiquitination and degradation^29^. Our findings revealed a novel and more universal function of TRIM29. Except for AM, TRIM29 was highly expressed in dsDNA-stimulated innate immune cells, such as BMDC and BMDM that have very low basal level of TRIM29. It is quite interesting that in BMDC and BMDM, TRIM29 did not response to LPS or poly (I:C) stimulation, which could be an explanation of why TRIM29 deficiency did not alter poly (I:C)-or LPS-stimulated cytokine production. So TRIM29 may regulate host immune defense by at two different mechanisms. First, the role of TRIM29 is regulated by its expression pattern. In the AM that is the first line of host defense in airway, constitutively highly expressed TRIM29 is required to maintain the local immune homeostasis. In the conventional innate immune cells such as DCs and macrophages, TRIM29 functions as the inducible regulator to mediate negative feedback and to prevent overcommitted immune response systemically. Second, the specificity of TRIM29 decides it functions in immune response. TRIM29 targets NEMO that is essential for NF-κB activation and also required for IFN-I production in RNA virus infection in AM. However, in DC and other macrophages, TRIM29 is induced by DNA virus and cytosolic dsDNA, and the substrate of TRIM29 is STING, which the crucial player in dsDNA-induced signal pathway. Together with the published results, our data suggest that TRIM29 is specific to catalyze K48 ubiquitination of its substrates, as TRIM29 induces the K48 ubiquitination and degradation of NEMO and STING in different settings.

TRIM29 is a member of TRIM E3 ligase family, lacking the typical RING domain. Instead, TRIM29 has two B-box domains, the structure, which is reported as catalytic motif with E3 ligase activity. TRIM18 has RING domain and B-box domain; both of them have E3 ligase activity although the B-box domain shows the weaker catalytic activity. We found that OmpH domain in the C-terminal of TRIM29 is required for the TRIM29–STING interaction; meanwhile this OmpH domain also is involved in TRIM29–NEMO interaction (through OmpH–OmpH interaction). These data are consistent with function of C-terminal of TRIM protein what is responsible for substrate interaction. The immunofluorescence results suggested that under the non-stimulated condition, overexpressed TRIM29 and STING sprayed in cytosol. HSV-60 stimulation caused the aggregation and translocation of STING around the nuclear, more importantly, TRIM29 also colocalized with STING perinuclearly, suggesting that TRIM29–STING interaction is induced by dsDNA stimulation.

**Fig. 6:**
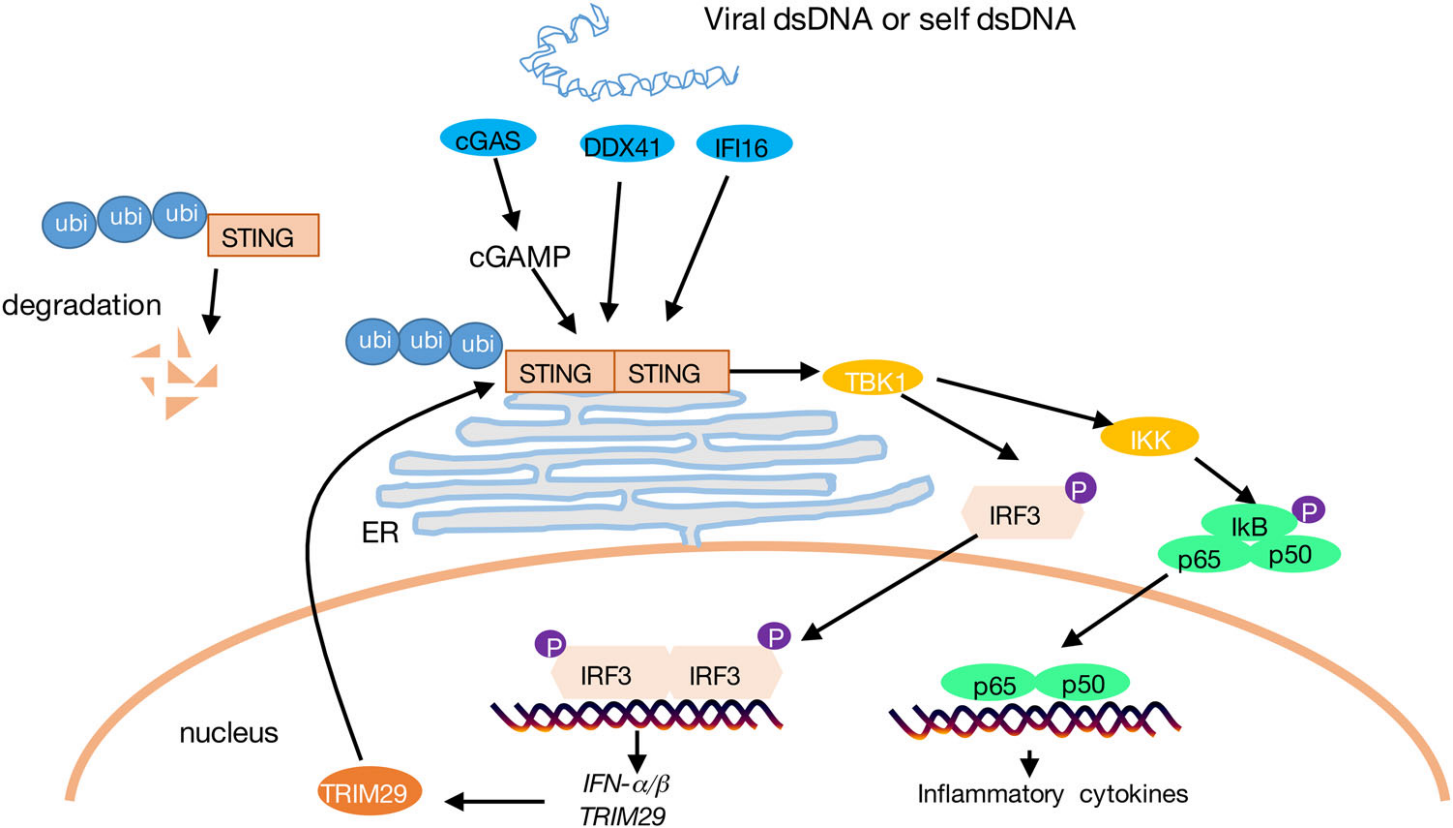
The schematic mechanism of TRIM29 regulating anti-DNA virus immune response through targeting STING. Viral dsDNA, or self-dsDNA, is sensed by cGAS and other DNA sensors such as DDX41 and IFI16. cGAS catalyzes the synthesis of cGAMP, which functions as a second messenger to activate STING–TBK1–IRF3 pathway, leading to IFN-I production. NF-κB pathway is also activated by TBK1 to trigger proinflammatory cytokine production. These cytokines are crucial to mediate an efficient antiviral immune response. TRIM29 is induced by DNA virus and cytosolic DNA stimulation, and targets STING for K48 ubiquitination and degradation, therefore downregulates STING-dependent signaling pathway, to prevent the overcommitted immune response

Collectively, our findings have established the crucial function of TRIM29 in antiviral immune response. TRIM29 response to DNA virus infection and cytosolic DNA stimulation, in turn mediates a negative feedback of STING–TBK1–IRF3 signaling pathway for IFN-I and proinflammatory cytokine production, and antiviral immune response in vivo (Fig. 6). Our study reveals a novel mechanism that the STING-dependent antiviral immune response is regulated by inducible negative regulator, which provides new strategy to improve vaccine development and to prevent self-DNA triggered autoimmunity.

## Materials and methods

### Mice

TRIM29 knockout mice were generated using CRISRP-Casp9 technique. The schematic diagram of knockout strategy was shown in Supp. Fig. 1a. Two guild RNA (gRNA) targeting the first exon of *Trim29* were used to deplete 265 bp from the first exon of *Trim29*, causing the degradation of truncated TRIM29 mRNA (Supp. Fig. 1a, 1b). Genotyping primers were used to amplify the 723-bp PCR product from the WT genomic DNA and 458-bp product was for *Trim29*^*-/-*^ genomic DNA (Supp. Fig. 1b). The mice were normal in development, without any appreciable abnormality. Real-time PCR and IB analysis result showed that TRIM29 was depleted in BMDM of *Trim29*^*-/-*^ mice in the mRNA and protein levels (Supp. Fig. 1c, 1d). The potential off-target sites based on the sequence of gRNAs were calculated, and sequencing results indicated that there was not any off-target site detected (Supp. Fig. 2a–2j). All animals were maintained in specific pathogen-free facility at the State Key Laboratory of Biotherapy, Sichuan University, China. Animal use and care were approved by the Animal Care Committee of the State Key Laboratory of Biotherapy, Sichuan University, in accordance with institutional animal care and use committee guidelines.

### Plasmids

Myc-Flag-tagged TRIM29 were from OriGene, different truncate TRIM29 mutants were cloned using the same vector, and mTRIM29 was cloned into pCDH-CMV-EF1-cop green fluorescent protein (GFP) (from OriGene) expression vector prepared for reconstruction of TRIM29. Hemagglutinin (HA)-tagged STING was cloned using pCMV6-Myc-Flag-STING (from OriGene) as template, and inserted into pCLXSN expression vector. STING mutants harboring K-to-R substitutions (K151R, K288R, and K337R and K288R/K337R) were generated by site-directed mutagenesis kit (Aligent) using pCMV6-Myc-Flag-STING as template, and confirmed by sequencing. WT and mutant ubiquitin vectors were described previously^36^.

TRIM29 shRNA and a control luciferase shRNA in pLKO.1 lentiviral vector were obtained from Open Biosystems.

### Antibody and reagent

The dsDNA derived from vaccinia virus, VACV-70, from herpes simplex virus type 1, HSV-60, cGAMP, LPS and poly (I:C) were from Invivogen. Lipofectamine 2000 was from Invitrogen. The proteasome inhibitor MG132 was from Sigma.

Rabbit polyclonal antibody for STING (161-378) was generated in Zen Bioscience, China; antibody for TRIM29 (SC-33151) was from Santa Cruz, antibodies for ubiquitin (04-263) and K48-specific anti-ubiquitin (05-1307) were from Millipore, antibody for β-actin (A2228) was from Sigma, anti-Myc (MA1-21316) was from Thermo. Anti-HA and anti-Myc beads were from Sigma. Horseradish peroxidase (HRP)-conjugated anti-HA antibody (ab183884) was obtained from Abcam, HRP-anti-Flag antibody (A8592) was from Sigma. Antibodies for phosph-TBK1 (5483), phosph-IRF3 (29047S), phosph-IkB (2859), total IRF3(4302), and total TBK1(3504) were purchased from Cell Signaling Technology. Fluorescein isothiocyanate (FITC)-goat anti-mouse IgG (ab6785) and Alexa Fluor 647-goat anti-rabbit IgG (ab150083) was from Abcam.

The mouse IFN-β and IFN-α enzyme-linked immunosorbent assay (ELISA) kit were from PBL InterferonSource. The mouse IL-6 and TNF-α ELISA kits were from eBioscience.

### Cell culture and stimulation

Culture of BMDM and BMDC were as described^29^. Briefly, the bone marrow was fleshed from femurs of adult mice (4–6 weeks old) and cultured in Dulbecco’s modified Eagle’s medium (DMEM) with 20% fetal bovine serum (FBS) plus 30% L929 conditional medium for BMDM differentiation, or in RPMI medium with granulocyte-macrophage colony-stimulating factor (10 ng/mL) for BMDC. The purity of BMDMs were determined by CD11b and F4/80 staining (the percentage of CD11b + F4/80 + population > 90%), and purify of BMDC was tested by CD11c (purity > 90%).

MEFs were prepared from embryos and cultured in DMEM with 10% FBS. For reconstruction of TRIM29 into *Trim29*^*-/-*^ MEF, HEK 293T cells were transfected with pCDH-CMV-mTRIM29-EF1-copGFP (or empty vector used as control) and lentivirus packaging plasmids. After 48 h, the supernatants were collected to infect *Trim29*^*-/-*^MEF cells in the presence of 8 μg/mL polybrene for 6 h. After another 48 h, GFP-positive cells were sorted by flow cytometry.

The cells were plated into 12-well or 6-well plates, and stimulated with different stimuli for further experiments.

For stimulation, HSV-60 (5 μg/mL), VACV-70 (5 μg/mL), dsDNA90 (5 μg/mL), 2′3′-cGAMP (1 μg/mL), LPS (100 ng/mL), and poly (I:C) (10 μg/mL) were used. The primers used to detect gene expression by qPCR were listed in Table 1.

**Table 1:** The sequence of primers used for qPCR

**Gene**	**Forward primer (5'–3')**	**Reverse primer (5'–3')**
*Trim29*	ACCTCCTGTGACCTTTGCTG	GGTAGCTATTCCTGCGGACT
*TRIM29*	CCTTCTCCCTGAAAGGCTATC	CGGTAGTGAGACAGCATAGAC
*Ifna*	TGACCTCAAAGCCTGTGTGATG	AAGTATTTCCTCACAGCCAGCAG
*Ifnb*	AGCTCCAAGAAAGGACGAACAT	GCCCTGTAGGTGAGGTTGATCT
*Il-6*	CACAGAGGATACCACTCCCAACA	TCCACGATTTCCCAGAGAACA
*Tnfa*	CATCTTCTCAAAATTCGAGTGACAA	CCAGCTGCTCCTCCACTTG
*Cxcl10*	CATCCTGCTGGGTCTGAGT	GATAGGCTCGCAGGGATGA
*Ccl5*	CACCATATGGCTCGGACAC	CGACTGCAAGATTGGAGCA
*Mx1*	GTACGGTGCAGACATACCAG	CGGTTTCCTGTGCTTGTATGA
*b-ACTIN*	CGAGGCCCAGAGCAAGAGAG	CGGTTGGCCTTAGGGTTCAG
*b-Actin*	CGTGAAAAGATGACCCAGATCA	CACAGCCTGGATGGCTACGT

### Ubiquitination assay

Total cell lysates were prepared and subjected to IB and co-IP assays as previously described^37^. Briefly, HEK 293T cells were transfected with expression vector of STING, HA-tagged ubiquitin and TRIM29 as indicated. Thirty-six hours after transfection, transfected cells were treated with 10 μM MG132 for 12 h. For endogens ubiquitination assay, cells were stimulated by HSV-60 or 2′3′-cGAMP with 10 μM MG132 for indicated time. Cells were lysed in RIPA buffer with 1% sodium dodecyl sulfate (SDS) and boiled for 5 min. After being diluted for 10 times with RIPA buffer lacking SDS, cell lysates were subjected to IP by anti-STING antibody. The ubiquitinated STING were detected by IB using an anti-ubiquitin antibody and anti-K48 ubiquitin antibody (for K48 ubiquitination).

### Viruses and in vivo infection

HSV-1 strain was propagated and titered by plaque assay on Vero cells as described previously^38^. Age- and gander-matched WT and *Trim29*^*-/-*^ mice (6- to 8-week old) were infected with HSV-1 (2 × 10^7^PFU per mouse) via tail vein injection. The survival rate of infected mice was monitored for 14 days. Sera were collected at indicated time points for ELISA analysis to detect IFN-α, IFN-β, IL-6, and TNF-α. Viral titers in brains, livers, and spleens of infected mice were measured by plaque assays. For plaque assay, briefly, viral samples were serially diluted and incubated with Vero in the plate for 1 h, the plate then was overlaid with 1.5% methylcellulose in minimum essential medium containing 1% FBS. Seventy-two hours later, cells were fixed in methanol and stained with 0.1% crystal violet. Plaques were counted to calculate viral titer.

### Confocal microscopy

HEK 293T cells were seeded on cover glass and transfected with expression vector of STING and Flag-tagged TRIM29. Forty-two hours after transfection, transfected cells were mocked (NT) or treated with HSV-60 for 6 h. The cells were washed with phosphate-buffered saline, fixed with 4% paraformaldehyde for 15 min and permeabilized with 0.5% Triton X-100 for 20 min at room temperature. After being blocked with 1% bovine serum albumin for 30 min at room temperature, the cells were incubated with anti-Flag antibody (mouse) and anti-STING antibody (rabbit) at 4 °C for overnight. The cells were further stained with secondary antibody FITC-anti-mouse IgG and AF647-anti-rabbit IgG, then examined with confocal microscopy.

### Mass spectrometric analysis of TRIM29-interacting proteins and STING ubiquitination sites

Raw 267.4 cells were stimulated for 6 h and TRIM29-interacting proteins were isolated by IP using anti-TRIM29 antibody. HEK 293T cells were transfected with pCMV6-Flag-TRIM29 and pCMV6-HA-STING with ubiquitin, with or without pcDNA-HA-ubiquitin, and the transfected STING was isolated by IP using anti-STING antibody. The conditions of cell lysate preparation and IP were the same as described for interacting protein assays and ubiquitination assays.

The isolated TRIM29-interacting proteins and STING proteins were subjected to mass spectrometric analysis in the lab of PTM at Sichuan University. In brief, the washed IP beads were boiled in 30 μl of 1X NUPAGE LDS sample buffer (Invitrogen) and subjected to SDS-polyacrylamide gel electrophoresis (PAGE) (NuPAGE 10% Bis-Tris Gel; Invitrogen). The eluted proteins were visualized with Coomassie Brilliant blue stain and excised into six gel pieces according to molecular size. The individual gel pieces were destained and subject to in-gel digestion using trypsin (GenDepot T9600). The tryptic peptides were resuspended in 10 μl of loading solution (5% methanol containing 0.1% formic acid) and subjected to nano Liquid chromatography-tandem mass spectrometry (LC-MS/MS) analysis with a nano-LC 1000 system (Thermo Fisher Scientific) coupled to Q Exactive plus (Thermo Fisher Scientific) mass spectrometer. The peptides were loaded onto an in-housed capillary Acclaim Pepmap 100 C18 column (75 μm ID × 15 cm length, 3 μm particle size, 100Å pore diameter, Dionex). The peptides were eluted in a 65-min linear gradient condition with solvent B (0.1% formic acid and 5% water in acetonitrile, v/v) from 6% to 90% in solvent A (0.1% formic acid and 2% acetonitrile in water, v/v) at a constant flow rate of 300 nl/min. Full-MS scans were acquired in the m/z range from 350.00 to 1800.00 with a resolution 70,000 at m/z 200. The top 20 precursors were selected for fragmentation with dynamical exclusion for 50 s. The isolation window and normalized collision energy were set to be 1.6 m/z and 27%, respectively.

Obtained MS/MS spectra of TRIM29-interacting protein and transformed STING were searched against MaxQuant software. All searches were performed against the SwissProt FASTA database (Musmusculus (mouse)) with trypsin as digestion enzyme. Alkylation of cysteine (+57.0215 Da) was specified as a fixed modification. Diglycine of lysine (+114.0429) for ubiquitination, oxidation of methionine (+15.9949 Da) and protein N-terminal acetylation (+42.0106 Da) were set as dynamic modifications. Twenty ppm was set for precursor and a maximum of two missed cleavages were allowed. The data were filtered with a false discovery rate ≤ 1% at both protein and peptide levels.

### Statistical analysis

Statistical analysis was performed using Prism software. Two tailed unpaired Student’s *t*-tests were performed. *P*-value < 0.05 were considered as statistically significant. The level of significance was indicated as *P < 0.05, **P < 0.01, ***P < 0.001. All statistical tests were justified as appropriate, and all data meet the assumption of test. The variance is similar between the groups being statistically compared^37^.

## Electronic supplementary material

Supplementary Information
