# Author Correction to: TRIM29 negatively controls antiviral immune response through targeting STING for degradation

**DOI:** 10.1038/s41421-018-0031-4

**Published:** 2018-05-31

**Authors:** Qijie Li, Liangbin Lin, Yanli Tong, Yantong Liu, Jun Mou, Xiaodong Wang, Xiuxuan Wang, Yanqiu Gong, Yi Zhao, Yi Liu, Bo Zhong, Lunzhi Dai, Yu-Quan Wei, Huiyuan Zhang, Hongbo Hu

**Affiliations:** 1 Department of Rheumatology and Immunology, State Key Laboratory of Biotherapy and Collaborative Innovation Center for Biotherapy, West China Hospital, Sichuan University, Chengdu, 610041 China; 2 Department of General Practice and Lab of PTM, State Key Laboratory of Biotherapy and Collaborative Innovation Center for Biotherapy, West China Hospital, Sichuan University, Chengdu, 610041 China; 3 Cancer Center, State Key Laboratory of Biotherapy and Collaborative Innovation Center for Biotherapy, West China Hospital, Sichuan University, Chengdu, 610041 China; 4 School of Life Science, Wuhan University, Wuhan, China

Correction to: *Cell Discovery* (2018) **4:13** 10.1038/s41421-018-0010-9, published online 20 March 2018

In the original publication of this article^1^ one of the author names was incorrect. In this correction article the correct and incorrect name are indicated.

Previous incorrect name:

Yu-Quan We

Corrected name:

Yu-Quan Wei

The original publication has been updated.
