# Supplementary material for: TRIM29 negatively controls antiviral immune response through targeting STING for degradation
Source: Cell Discov. 2018 Mar 20;4:13. doi: 10.1038/s41421-018-0010-9 (PMC5859251; doi:10.1038/s41421-018-0010-9)
Supplement: Supplementary file 1 — Supplementary Information [file 41421_2018_10_MOESM1_ESM.pdf]

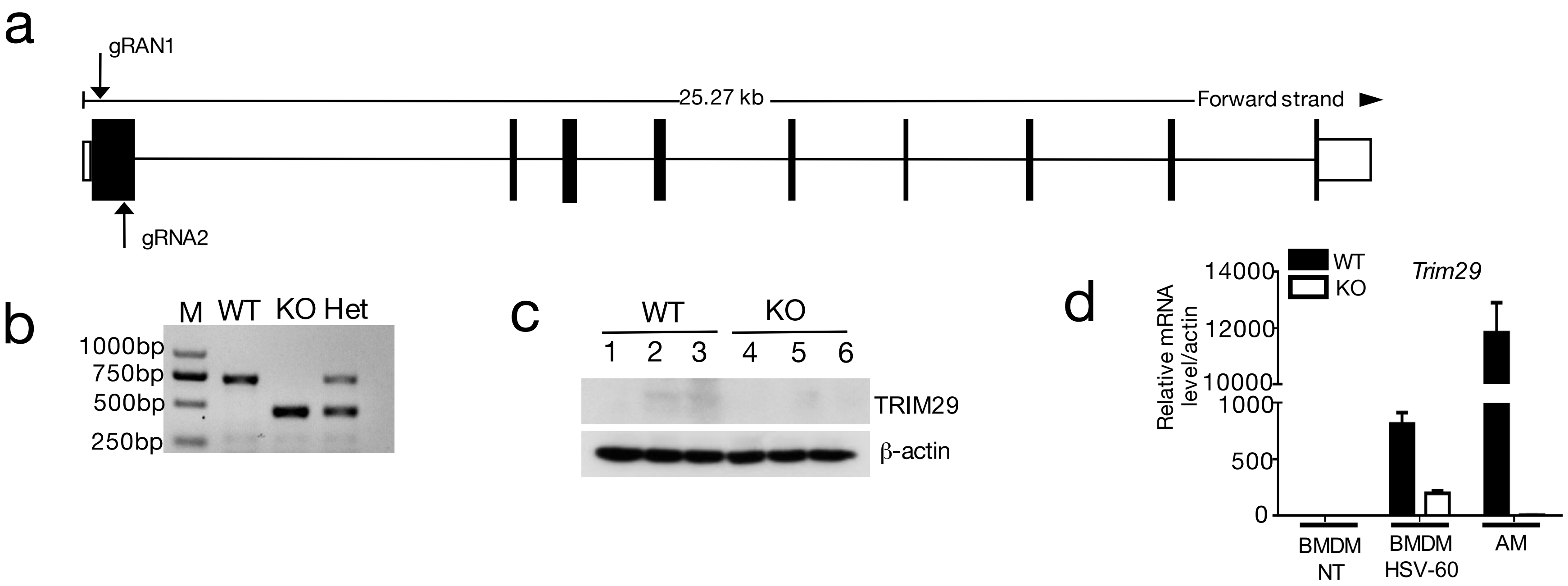

Supp. Figure 1 The generation of *Trim29* KO mice using CRISPR-Cas9 technology

TRIM29 knockout mice were generated using CRISPR-Cas9 technology. The schematic diagram of knockout strategy was showed in **Supp.Fig1a**. Two gRNA targeting the first exon of *Trim29* were used to induce gene editing of TRIM29, causing depletion of 265bp of the first TRIM29 and degradation of TRIM29 mRNA (**Supp. Fig1a,1b**). Primers were designed for genotyping, resulting in a 723bp for WT and 458bp for KO (Supp.Fig1b). The mice were normal in development, without any appreciable abnormality. Immunoblot assay (**Supp. Fig.1c**) and qPCR (**Supp. Fig.1d**) result showed that TRIM29 was depleted in HSV-60-stimualted-BMDM and alveolar macrophage of *Trim29* KO mice. Line 1: WT BMDM, non-treated; line2: WT alveolar macrophage; line 3: WT BMDM, HSV-60 stimulated for 24hrs; Line 4: KO BMDM, non-treated; line5: KO alveolar macrophage; line 6: KO BMDM, HSV-60 stimulated for 24hrs.

a, Chr 16
41327216 to 41327238
TGACCTGGGCCACTCATTGCAAG

b, Chr 2
51130478to 51130500
ATCCCTGCCCCACTCATTGCTAG

c, Chr 6
123340959 to 123340981
AGCCCTGGGCCATTCATTGCTAG

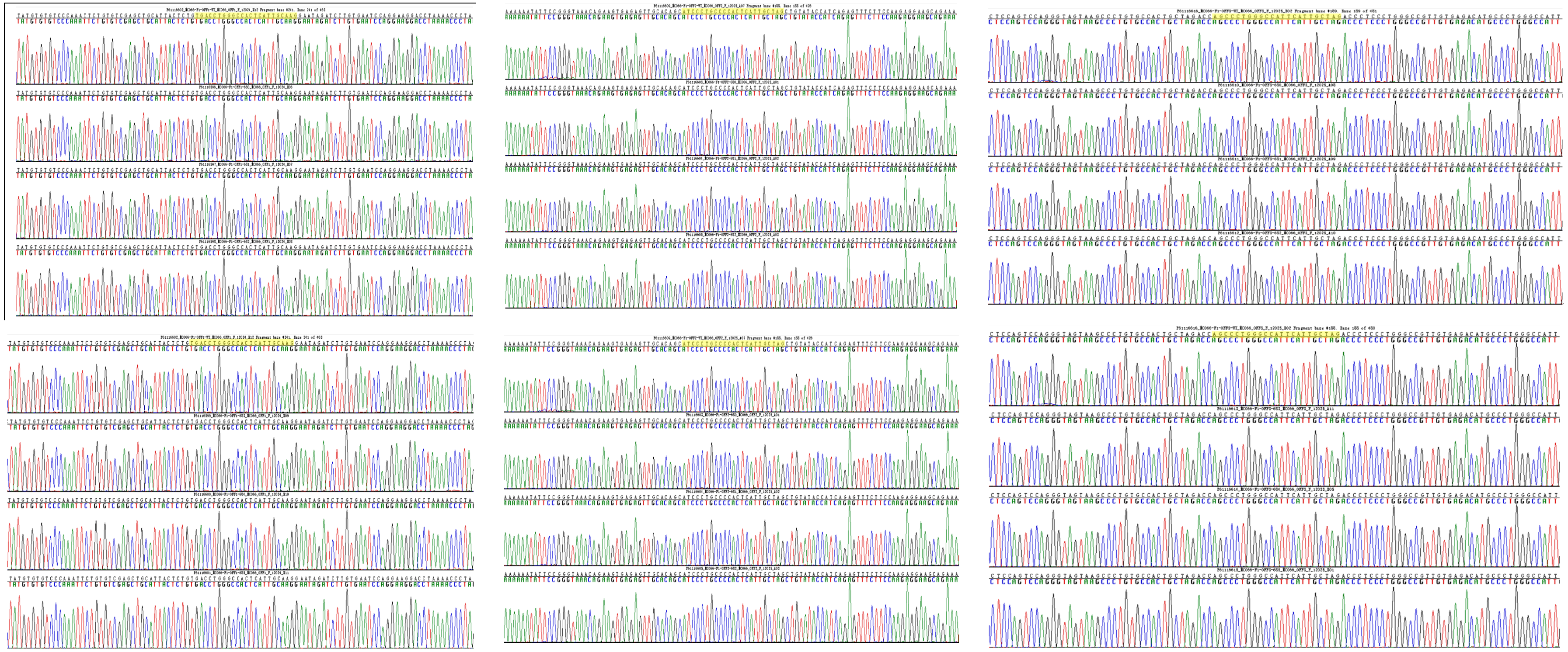

Supp. Figure 2 Sequencing results of potential off-target sites of CRISPR-Cas9

The potential off-target sites were examined by sequencing **(Supp. Fig. 3a-3j)**.

d, Chr 8
33528824 to 33528846
AGTGCTGCTACACTCATTGCAGG

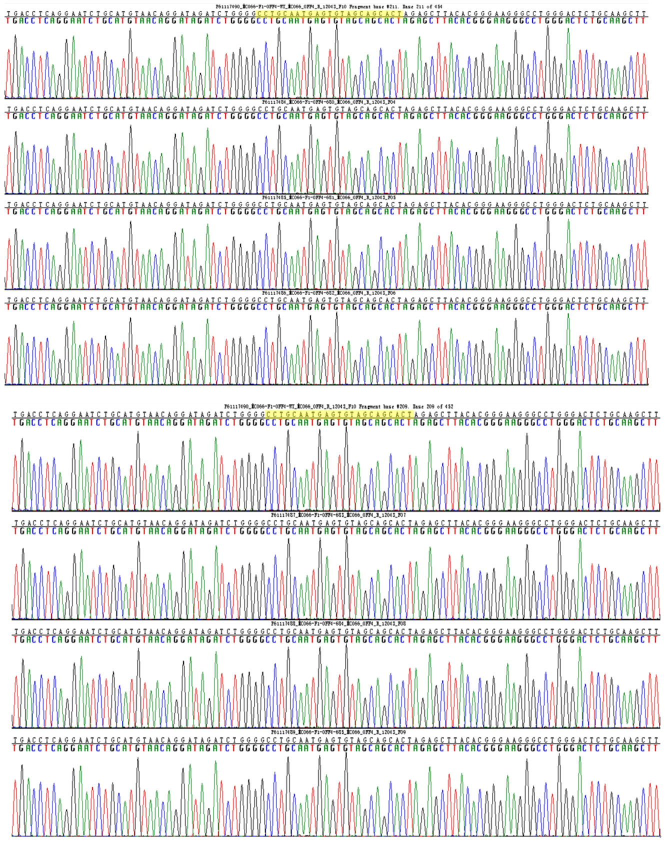

e, Chr 9
39791494 to 39791486
AGACCTGTGGCACTCATTGAGGG

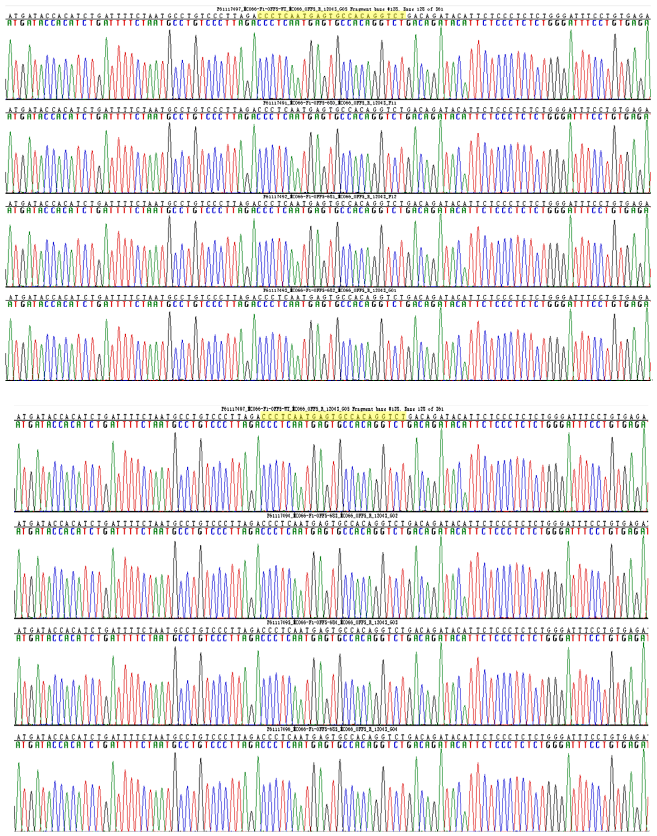

f, Chr 11
73392652 to 73392674
TCCTCTTTCCCCGCGCCAGGGGG

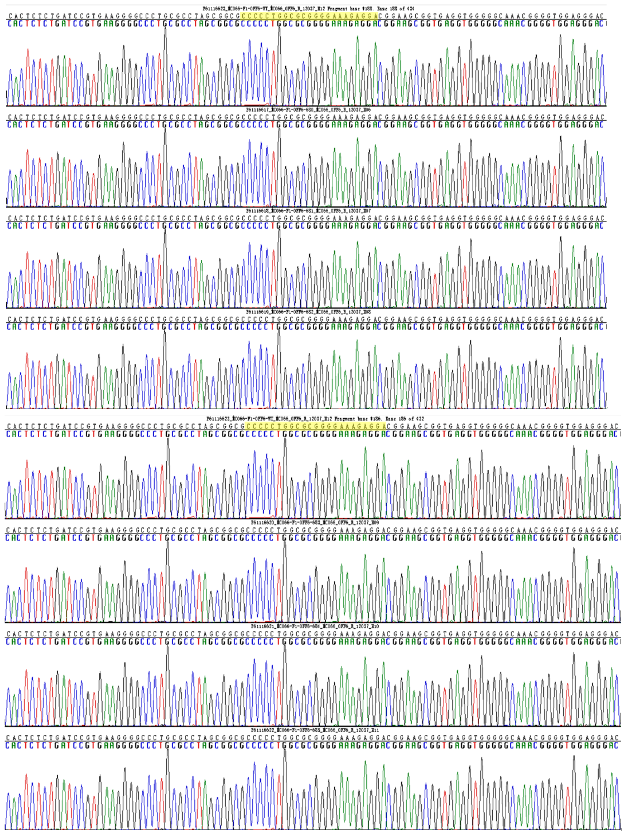

Supp. Figure 2 Sequencing results of potential off-target sites of CRISPR-Cas9

The potential off-target sites were examined by sequencing **(Supp. Fig. 3a-3j)**.

g, Chr 4
166139465 to 166139487
CAGGCTCTTCCCGCGCCAGGCAG

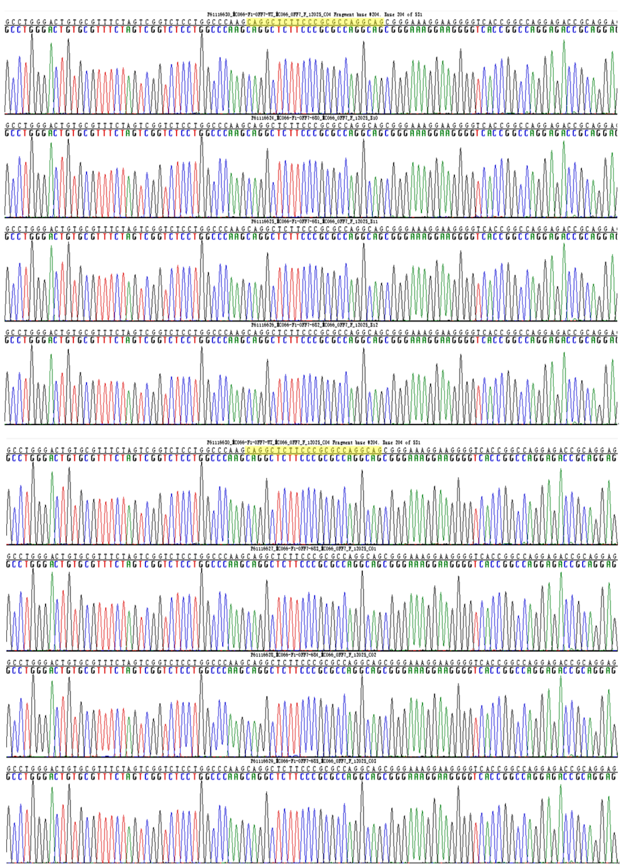

h, Chr 15
82077028 to 82077050
CCGAATCTGCCCGCCCCAGGAGG

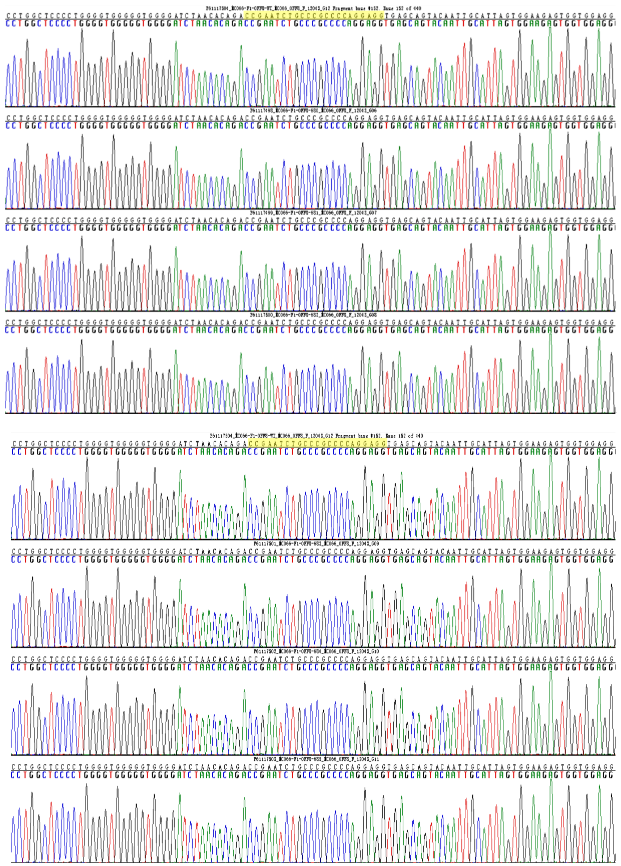

i, Chr 5
147397771 to 147397793
CCGGCTTTGCCAGCTCCAGGCGG

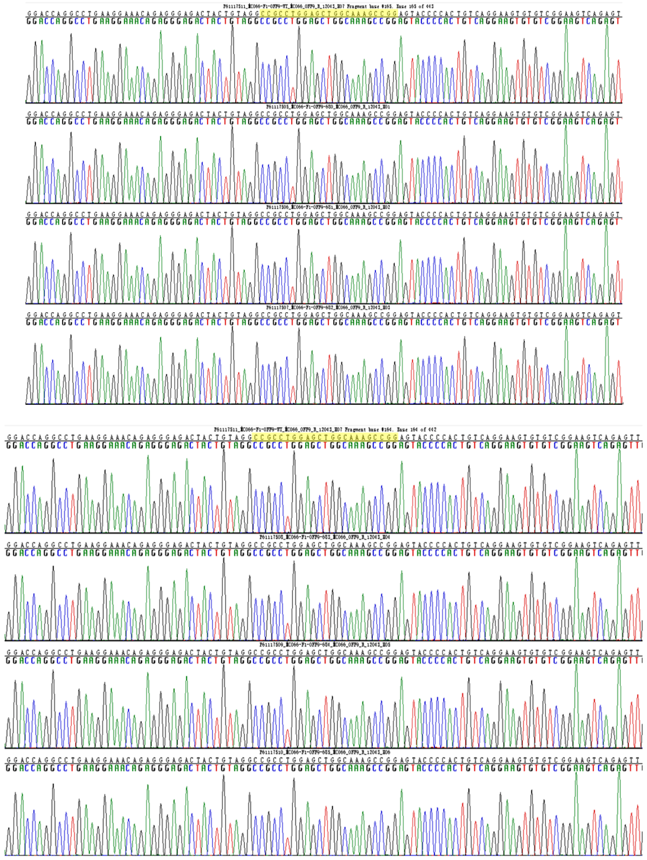

Supp. Figure 2 Sequencing results of potential off-target sites of CRISPR-Cas9

The potential off-target sites were examined by sequencing **(Supp. Fig. 3a-3j)**.

j, Chr 1
65631761 to 65631783
GAGACTCTGCCCCGCTCCAGGCGG

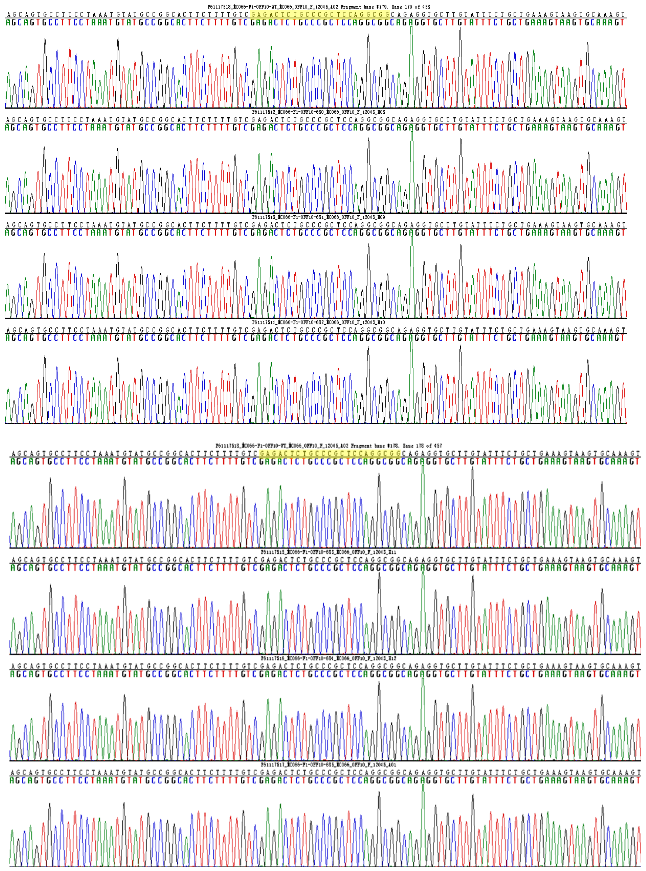

Supp. Figure 2 Sequencing results of potential off-target sites of CRISPR-Cas9

The potential off-target sites were examined by sequencing **(Supp. Fig. 3a-3j)**.

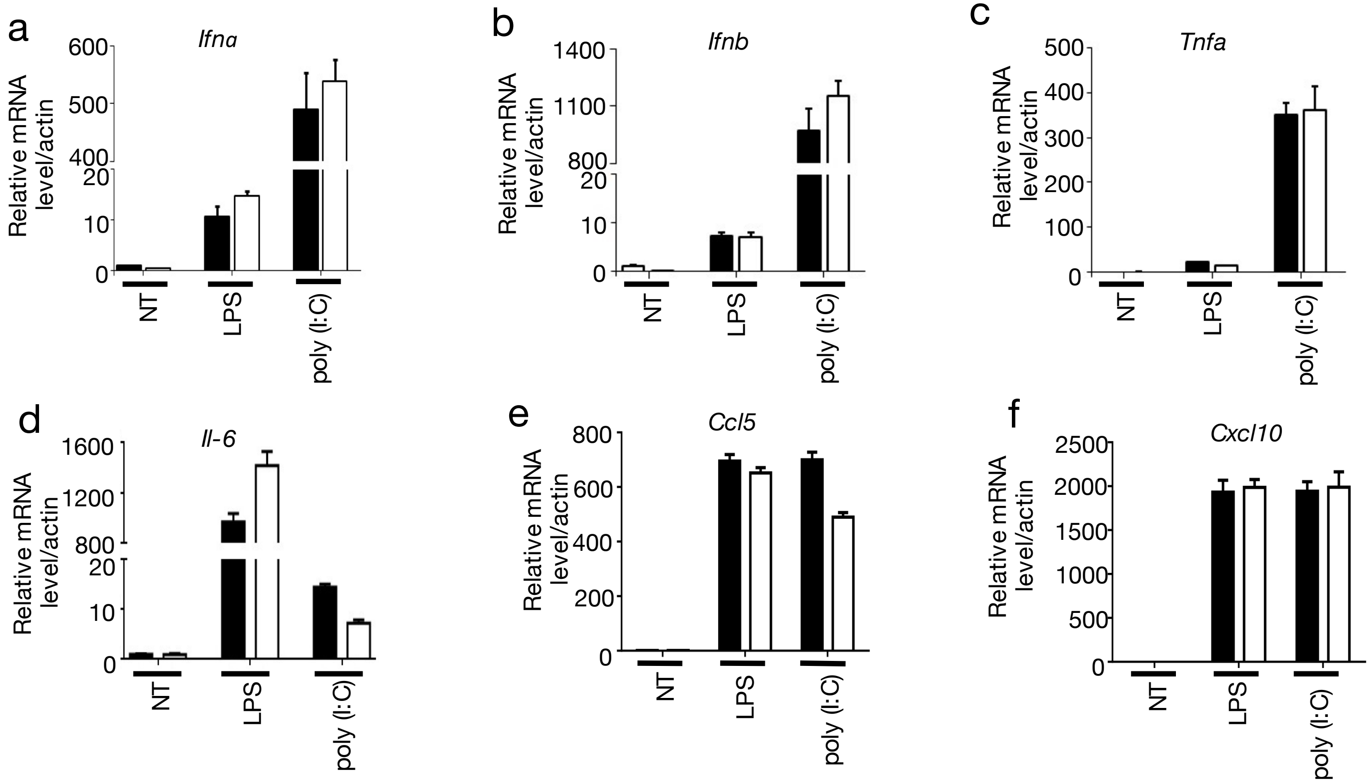

Supp. Figure 3 TRIM29 is dispensable for cytokine, chemokine production induced by LPS and poly I:C

WT and TRIM29 KO BMDMs were stimulated with LPS and poly I:C for 12 hrs. The mRNA level of proinflammatory cytokines, chemkines and antiviral protein was detected by qPCR.

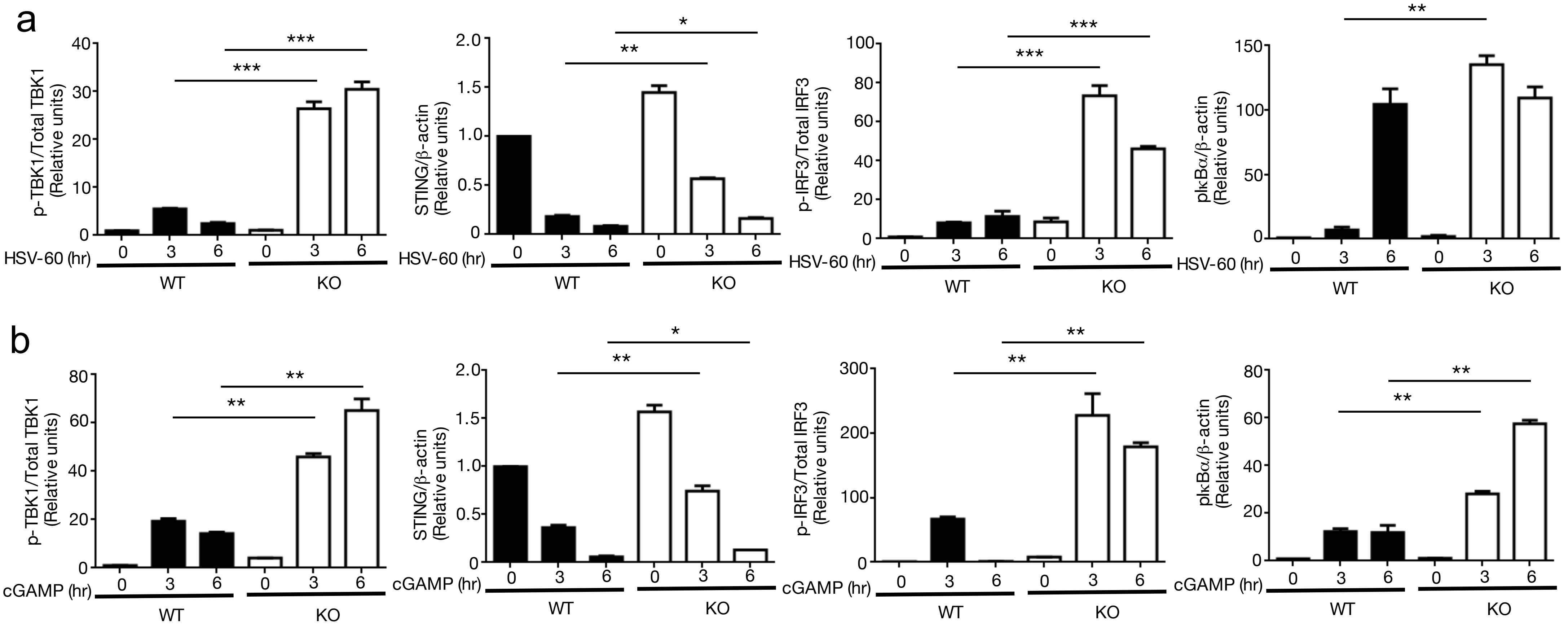

Supp. Figure 4 The relative density of western blot result in figure 3a

WT and TRIM29 KO BMDMs were stimulated with HSV60 (a) and cGAMP (b) for indicated time. The phosphorylation of TBK1, IRF3, IκBα, and the degradation of STING detected by WB. The total proteins or β-actin served as loading control. The relative density of bands in figure 3a were measured using Image J for three times. The activations of TBK1, IFR3 and IκBα were indicated by ration of phospho-protein vs total protein or β-actin, the degradation STING was shown as the ration of STING vs β-actin.

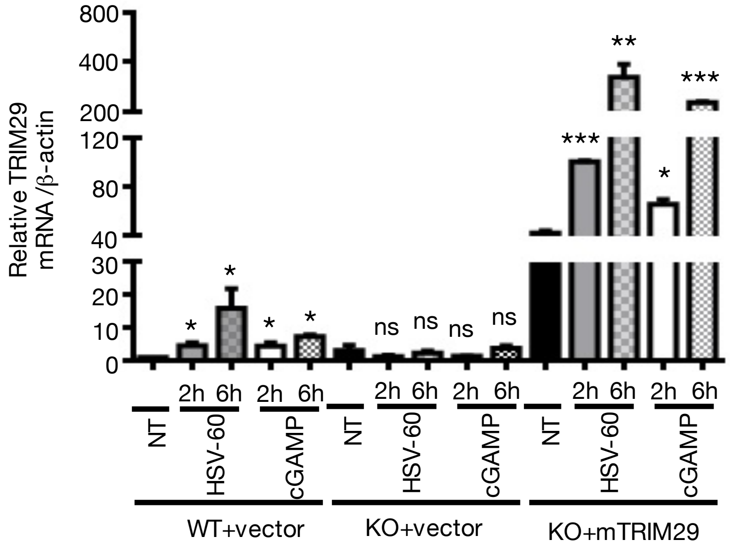

Supp. Figure 5 TRIM29 mRNA is quickly induced by dsDNA stimulation in both WT and *Trim29*-reconstituted MEF

WT, *Trim29*-/- mice and *Trim29*-reconstituted MEF were stimulated with HSV-60 or cGAMP for 0,2,6 hrs. The expression of TRIM29 mRNA was detected by real-time PCR and β-actin served as the reference gene.

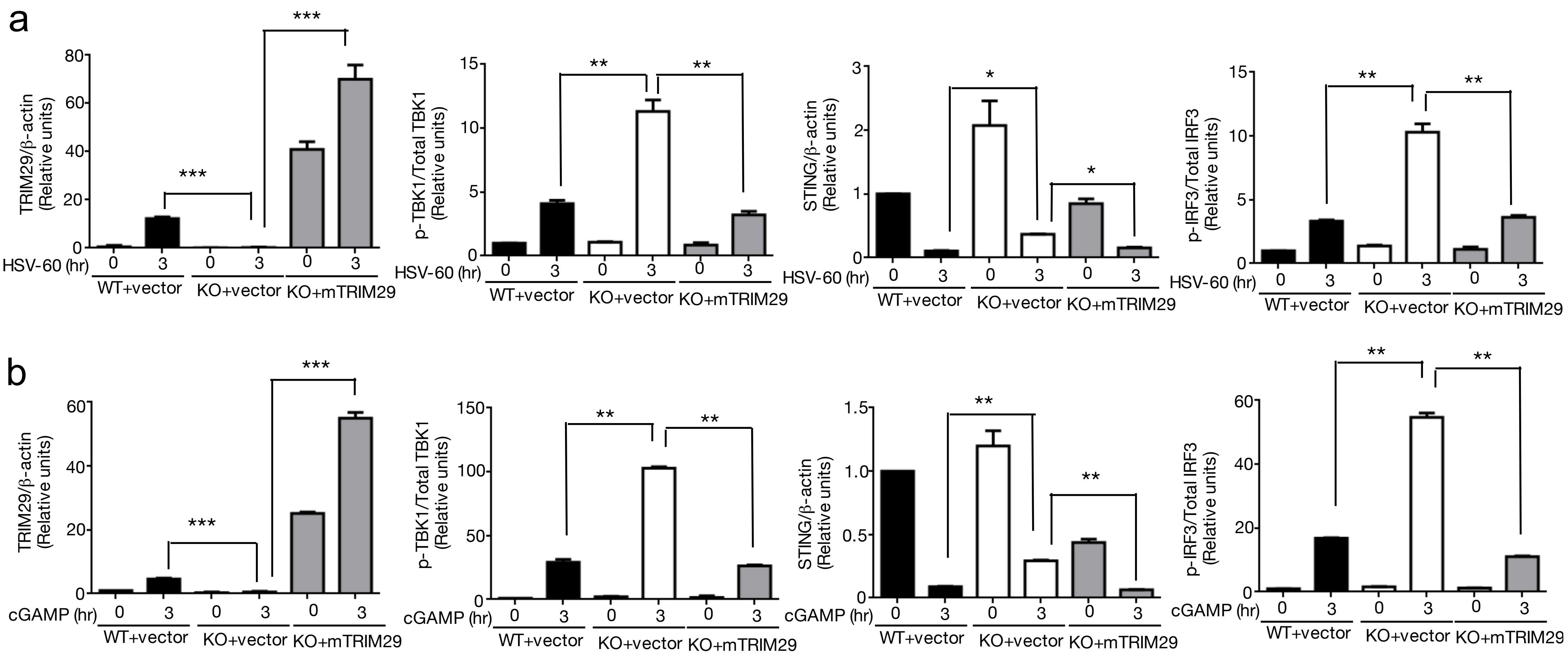

Supp. Figure 6 The relative density of western blot result in figure 3b

WT, *Trim29*$^{-/-}$ and TRIM29-reconstructited MEFs were stimulated by HSV-60 (a) and cGAMP (b) for indicated time, and IB was performed to detect TRIM29, STING, phosphorylation of TBK1, IRF3. The total proteins or β-actin severed as loading control. The relative density of bands in figure 3b measured using Image J for three times. The activations of TBK1 and IFR3 were indicated by ration of phospho-protein vs total protein, the degradation STING was shown as the ration of STING vs β-actin.

Fig.4

a

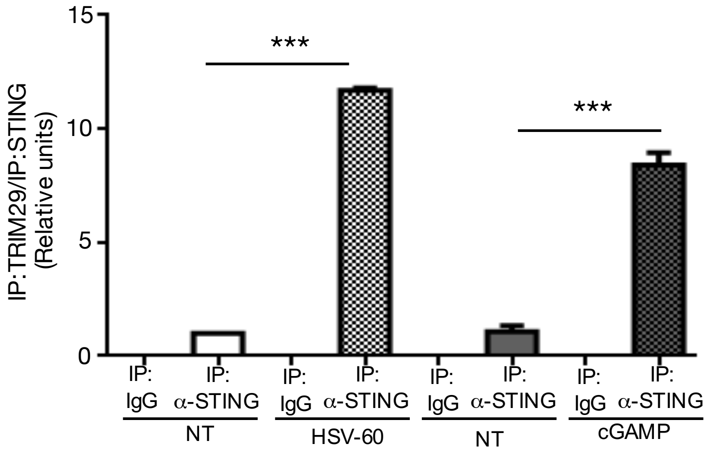

b

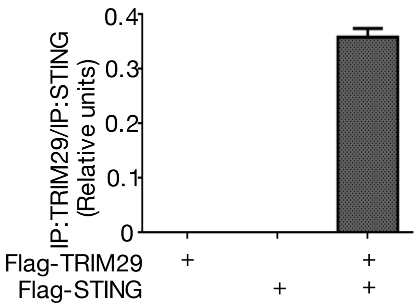

Supp. Figure 7 The relative density of western blot result in figure 4b and 4c

The immunoprecipitation experiments were described in Fig. 4b and Fig.4c. The relative density of bands in Fig.4b and Fig. 4c measured using Image J for three times. The interaction of endogens (a) and overexpressed (b) TRIM29 and STING were indicated by rations of precipitated TRIM29 vs precipitated STING.

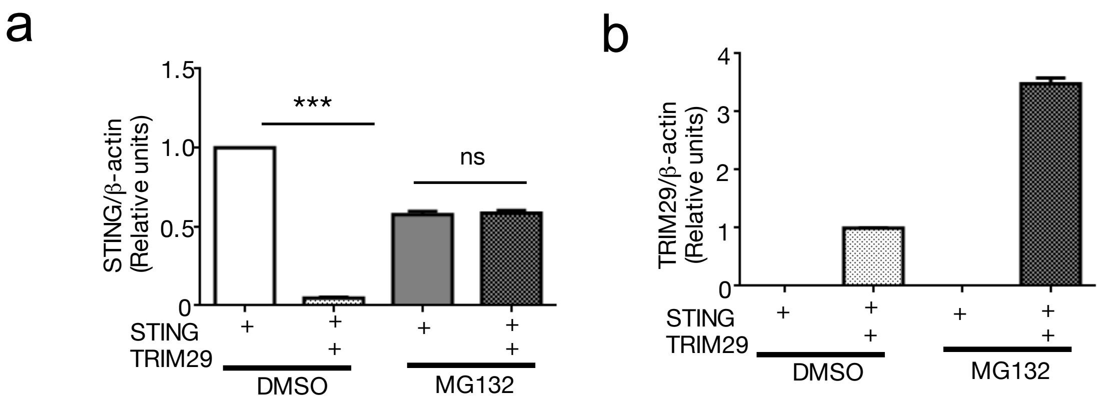

Supp. Figure 8 The relative density of western blot result in figure 4e

TRIM29 and STING were overexpressed in 293T cell, treated with DMSO or MG132. The expression of TRIM29 and STING detected by WB (Fig.4e). The relative density of STING, TRIM29 and β-actin in Fig.4e measured using Image J for three times. The rations of STING/β-actin (a) and TRIM29/β-actin (b) were calculated.

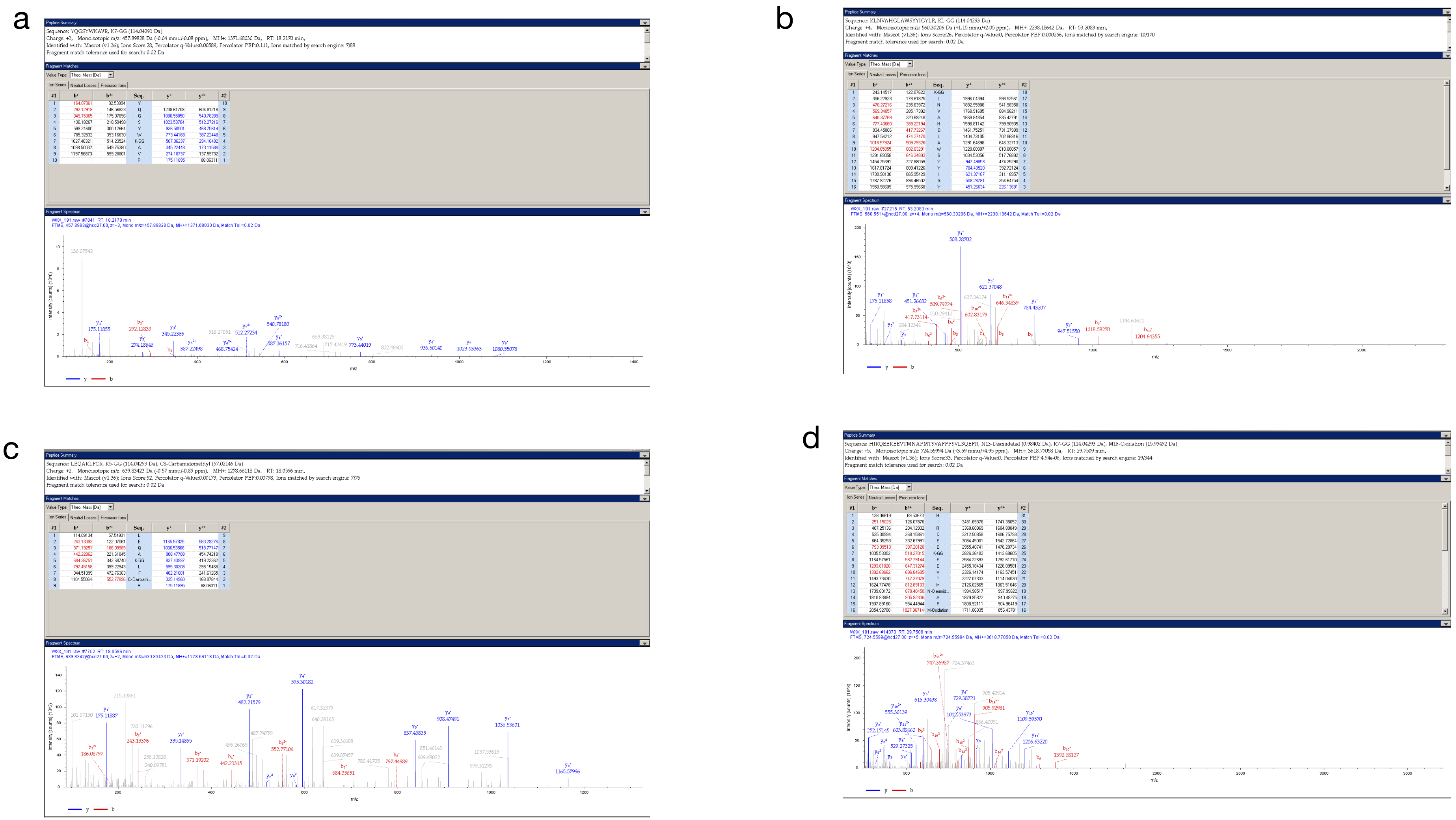

Supp. Figure 9 MS results of identification of Lys (K) in STING targeted by TRIM29

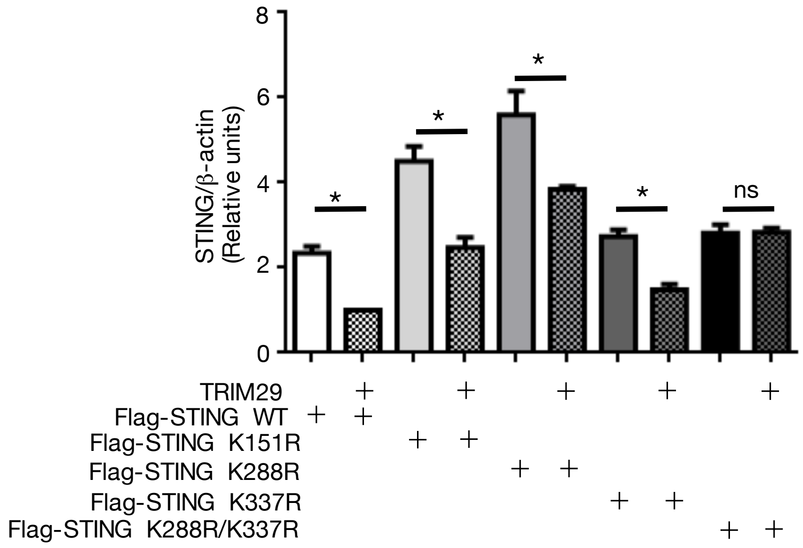

Supp. Figure 10 The relative density of western blot result in figure 5h

WT or mutant STING were overexpressed in 293T cell, with or without TRIM29. The expression of WT and mutant STING detected by WB (Fig.5h). The relative density of STING and β-actin in Fig.5h measured using Image J for three times. The rations of STING/β-actin were calculated.
